# Ferroptosis inhibitors: past, present and future

**DOI:** 10.3389/fphar.2024.1407335

**Published:** 2024-05-23

**Authors:** Lei Zhang, Yi Lin Luo, Yang Xiang, Xin Yue Bai, Rong Rong Qiang, Xin Zhang, Yan Ling Yang, Xiao Long Liu

**Affiliations:** ^1^ School of Medicine, Yan’an University, Yan’an, China; ^2^ College of Physical Education, Yan’an University, Yan’an, China

**Keywords:** ferroptosis, inhibitors, iron metabolism, lipid metabolism, antioxidants system, clinical translation

## Abstract

Ferroptosis is a non-apoptotic mode of programmed cell death characterized by iron dependence and lipid peroxidation. Since the ferroptosis was proposed, researchers have revealed the mechanisms of its formation and continue to explore effective inhibitors of ferroptosis in disease. Recent studies have shown a correlation between ferroptosis and the pathological mechanisms of neurodegenerative diseases, as well as diseases involving tissue or organ damage. Acting on ferroptosis-related targets may provide new strategies for the treatment of ferroptosis-mediated diseases. This article specifically describes the metabolic pathways of ferroptosis and summarizes the reported mechanisms of action of natural and synthetic small molecule inhibitors of ferroptosis and their efficacy in disease. The paper also describes ferroptosis treatments such as gene therapy, cell therapy, and nanotechnology, and summarises the challenges encountered in the clinical translation of ferroptosis inhibitors. Finally, the relationship between ferroptosis and other modes of cell death is discussed, hopefully paving the way for future drug design and discovery.

## Graphical Abstract

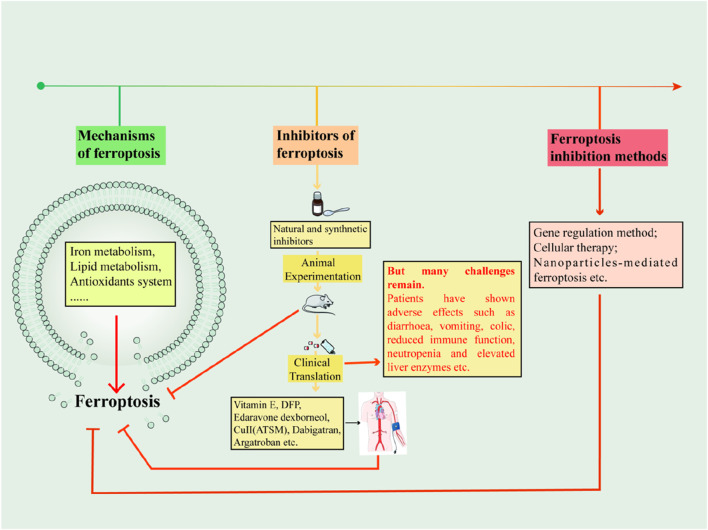

## 1 Introduction

Ferroptosis was first observed in 2003 and officially named by Dixon et al., in 2012. Ferroptosis is a programmed cell death characterized by iron dependence and lipid peroxidation (Chen et al., 2021). Ferroptosis is morphologically and biochemically distinct from traditional modes of cell death such as necrosis, apoptosis, and autophagy. Morphologically, ferroptosis is mostly characterized by smaller mitochondria, increased membrane density, reduced or absent mitochondrial ridges, and normal cell size but a lack of chromatin cohesion (Sun et al., 2023). Biochemically, large amounts of unsaturated fatty acids in the cell membrane undergo lipid peroxidation in response to Fe^2+^ or lipoxygenase (LOX), triggering ferroptosis (Tang et al., 2021). Ferroptosis mainly involves three pathways: iron metabolism, lipid metabolism, and antioxidants system. Disturbed iron metabolism triggers the Fenton reaction and induces the accumulation of ROS, accumulation of lipid peroxides, and insufficient Glutathione Peroxidase 4 (GPX4) leading to the difficult conversion of lipid hydroperoxides are at the core of the ferroptosis induced by iron metabolism disorders.

With the proposal of ferroptosis, ferroptosis has been found to mediate the pathogenesis of many diseases. In recent years, it has been found that ferroptosis inhibitors also play an important role in neurodegenerative diseases (e.g., stroke (Millán et al., 2021), Alzheimer’s disease (AD) (Jakaria et al., 2021), spinal cord injury (SCI) (Yu et al., 2023)), Acute kidney injury (AKI) (Guerrero-Mauvecin et al., 2023). Intervening in ferroptosis may therefore lead to new therapeutic strategies for the disease. Several drugs have been reported to play an inhibitory role in iron-death-mediated diseases. This review systematically describes the metabolic pathways of ferroptosis and summarizes the mechanisms of action of natural and synthetic small-molecule inhibitors of ferroptosis as well as their applications in disease. Non-traditional therapeutic approaches such as gene therapy, cell therapy, drug combinations and nano-delivery in ferroptosis-mediated diseases are also presented. In addition, the challenges encountered in the preclinical experimental stage and clinical translation of ferroptosis inhibitors are summarised. Finally, the relationship between ferroptosis and other modes of cell death is discussed in the hope of providing new ideas for future drug design and development and clinical translational applications.

## 2 Small molecule ferroptosis inhibitors

### 2.1 Inhibition of ferroptosis via the iron metabolism pathway

**FIGURE 1:**
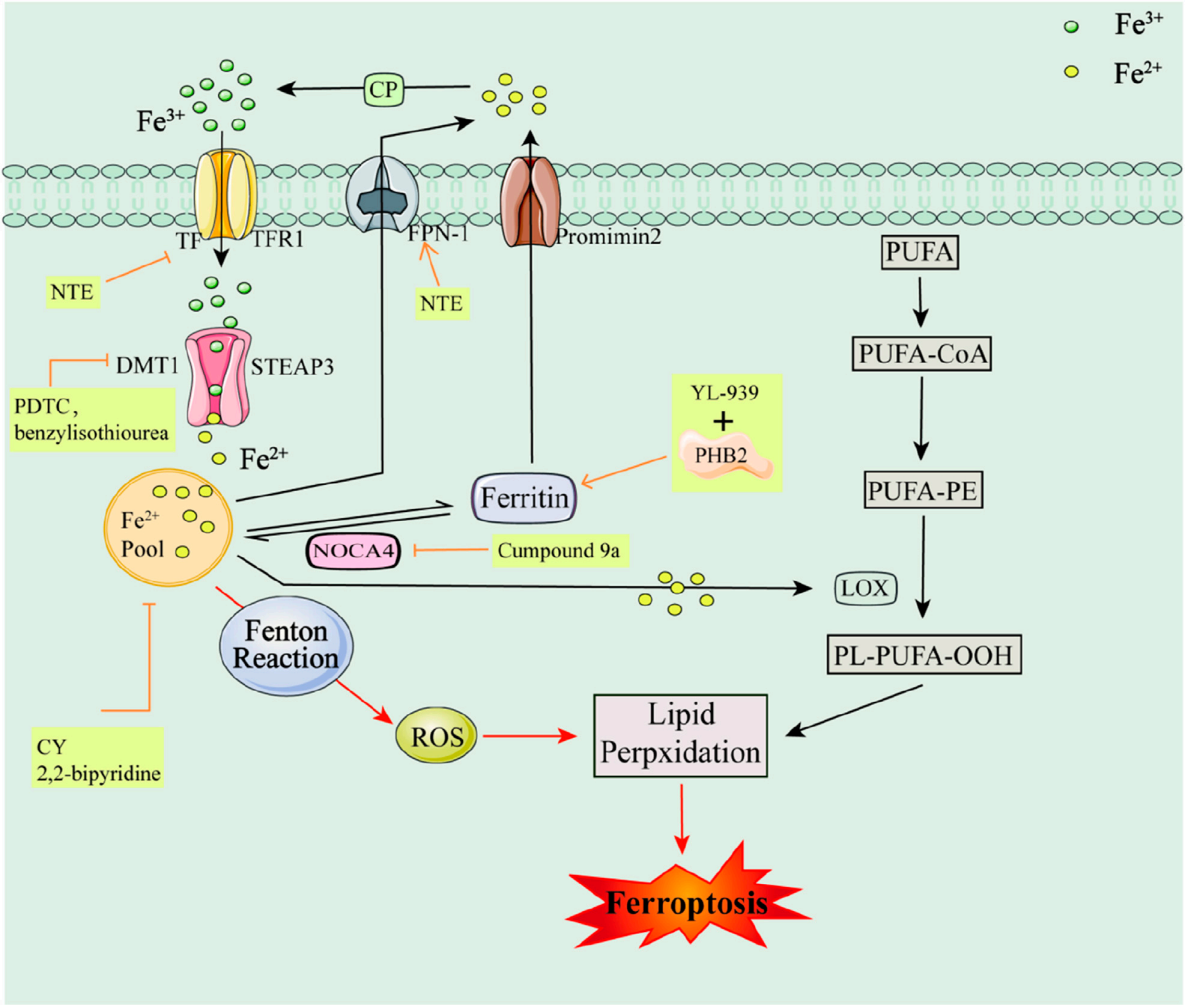
Iron metabolism pathways and their associated inhibitors of ferroptosis Serum Transferrin (TF); The membrane protein Transferrin Receptor1 (TFR1); Six-Transmembrane Epithelial Antigen of Prostate 3 (STEAP3); Ceruloplasmin (CP); Divalent Metal Transporter 1 (DMT1); Polyunsaturated fatty acids (PUFAs); Phosphatidyl Ethanolamine-Polyunsaturated Fatty Acids (PE-PUFA); Lipoxygenase (LOX); Ferritin; Membrane iron transport protein 1 (Ferroportin1, FPN1); Nuclear receptor coactivator 4 (NCOA4); Lipid hydroperoxide (PL-PUFA-OOH); Promimin 2.

Iron is one of the important trace elements in the human body and is a key causative factor in ROS accumulation and ferroptosis. Under normal conditions, Fe^3+^ is found in serum transferrin (TF), The membrane protein Transferrin Receptor1 (TFR1), Six-Transmembrane Epithelial Antigen of Prostate 3 (STEAP3) enzyme, Divalent Metal Transporter 1 (DMT1), Nuclear Receptor Co-Activator 4 (NRC4), and the serum transferrin (TF) enzyme, and in the serum transferrin (TF) and TFR1 enzymes. Epithelial Antigen of Prostate 3 (STEAP3) enzyme, Divalent Metal Transporter 1 (DMT1), and Nuclear receptor coactivator 4 (NCOA4) (Gryzik et al., 2021), Membrane iron transport protein 1 (Ferroportin1, FPN1), Ferritin, Promimin 2, Ceruloplasmin (CP), etc. are involved in the transport to cells and participate in the reaction *in vivo* (Scarpellini et al., 2023; Zhang et al., 2024a). However, the excess of Fe^2+^ in the organism under certain pathological conditions, on the one hand, induces the accumulation of ROS in the body through the Fenton reaction; on the other hand, iron participates in the catalytic process of metabolic enzymes, such as LOX, as a cofactor of various phospholipid peroxidases, which in turn accelerates lipid peroxidation and induces ferroptosis (Du and Guo, 2022; Sun et al., 2023) (e.g., Figure 1). Therefore, regulating Fe^2+^ levels in the body could potentially inhibit ferroptosis and aid in disease treatment.

#### 2.1.1 Iron chelators

**Deferoxamine (DFO)** has been approved by the Food and Drug Administration (FDA) for subcutaneous injection to mitigate elastin-induced ferroptosis in an *in vitro* ferroptosis model (Abdul et al., 2021). **DFO** inhibits ferroptosis by chelating free intracellular Fe^3+^, down-regulating ROS, and up-regulating intracellular levels of GPX4, the ferritin heavy chain (FTH1), and Cystine/glutamic acid reverse transporter (System Xc-) (Zhang et al., 2020; Zeng et al., 2021). Moreover, **DFO** has been shown to inhibit ferroptosis in SCI (Yao et al., 2019), but whether it directly protects neurons from ferroptosis is unclear. At the same time, **DFO** also plays a role in ischemic stroke (IS), which significantly reduces the area of cerebral infarction (Millán et al., 2021), and plays a certain neuroprotective role against the damage of neurological function after cerebral ischemia (Jones et al., 2022). However, a short **DFO** half-life was found during treatment. At the same time, two oral drugs, **Deferiprone (DFP)** and **Deferasirox (DFX)**, have been developed to address the short half-life of **DFO**, but side effects such as granulocyte deficiency (Lecornec et al., 2022)and renal failure (Kattamis, 2019)are still present during treatment. To address the limitations of **DFO**, **DFP**, and **DFX** such as low oral activity, low efficacy, and side effects, Chen et al. introduced a sacrificial site for glucuronidation and designed and synthesized a novel oral iron chelator, **CN128**, and found that **CN128** was more effective and efficacious orally (Chen et al., 2020), and it has been used in clinical trials in β-thalassemia patients after regular blood transfusions.

Deferric Amine Compounds **DFA1** bind iron through two molecules of oxygen in the phenolic hydroxyl group and one molecule of nitrogen in the amine. Research has shown that **DFA1** efficiently chelates iron *in vivo* and *ex vivo*, outperforming **DFO**. It has also been observed to alleviate iron overload-induced ferroptosis and reduce cytotoxicity in a mouse model of iron overload when administered orally and intravenously (Feng et al., 2022). Furthermore, **DFA1** shows promise as a lead compound due to its high oral (Feng et al., 2022).

**DFP** was shown to have nephroprotective effects in a glycerol-induced AKI mouse model. Zhang et al. synthesized 25 novel iron chelator cinnamamide-hydroxypyridone derivatives by combining the iron chelating properties of **DFP** with the free radical scavenging capabilities of phenolic acids. Their assessment included assays for ABTS radical scavenging, Fe^3+^ affinity, oxygen radical absorbance capacity (ORAC), and the inhibition of Erastin-induced ferroptosis in HT22 cells. It was shown that compound **9c** has both chelating iron ions and antioxidant properties. **Compound 9c** exhibited the strongest inhibition of ferroptosis, almost 10 times more potent than **DFP** (EC_50_ = 14.89 μM). Additionally, the researchers observed that **compound 9c** significantly alleviated cisplatin (CP)-induced AKI in the HEK293T cell model (Rayatpour et al., 2022).

**Dexrazoxane (DXZ)** is the only drug approved by the FDA that can be used to prevent doxorubicin (DOX)-induced cardiotoxicity. **DXZ** has been reported to reverse DOX-induced ferroptosis mainly by chelating mitochondrial iron, and its co-administration with **Ferrostatin-1 (Fer-1)** increased survival in cardiomyopathic rats. Moreover, DOX upregulated GPX4 as well as FTH1 in H9c2 cells (Zhang et al., 2021; Huang et al., 2024). Meanwhile, studies have reported that **2,2′-Bipyridine** exerts ferroptosis inhibition by chelating intra-mitochondrial iron (Chen et al., 2020). **1,10-Phenanthroline** resembles and downregulates mitochondrial ROS accumulation and inhibits ferroptosis induced by zero-valent iron nanoparticles *in vitro* with **2,2′-Bipyridine** (Huang et al., 2019; Chen et al., 2020). Furthermore, the N, N-dimethylaniline structure of the novel iron chelator **GIF-2197-r** is crucial for ferrous ion coordination and ferritin resistance (Hirata et al., 2023). In addition, Yael Avramovich-Tirosh et al. reported that the synthetic iron chelator **[5-(N-methyl-N-propargylaminomethyl)-8-hydroxyquinoline] (M-30)**, which permeates the BBB and reduces cellular APP and Aβ production levels, was shown to alleviate the intellectual disability in mice after AD in an *in vivo* animal model (Avramovich-Tirosh et al., 2007; Zhang et al., 2022).

In addition, studies have shown that natural compounds also can chelate iron. The flavonoid **baicalein** significantly reverses elastin-induced downregulation of iron accumulation, GSH, and GPX4 in cells (Xie et al., 2016). Experimental screening yielded **Hinokitiol**, a natural molecule carrying an α-hydroxy ketone skeleton with strong iron chelating properties, which can activate nuclear factor red factor 2-related factor 2 (Nrf2), providing a basis for further neuroprotection (Sridharan and Sivaramakrishnan, 2018). **Tannins (TA)**, which complex with iron without binding to endogenous iron-containing molecules, are also an effective measure for the treatment of diseases associated with iron overload (Phiwchai et al., 2018). **BMS536924** a dual inhibitor of insulin-like growth and insulin receptor protects against the induction of iron-dead cells and can act as an iron chelator to block ferroptosis (Kuganesan et al., 2021). **Thymus β4** is an endogenous iron chelator that regulates ferroptosis by affecting free iron ions and ROS (Lachowicz et al., 2022). **Ciclopirox (CPX)** (Eberhard et al., 2009; Lin J. et al., 2021; Lu et al., 2022) has been approved by the FDA for antifungal therapy, and in recent years it has been found that intraperitoneal injection can significantly inhibit the growth of non-small cell lung cancer (NSCLC). Therefore, the use of iron chelation therapy in clinical practice still holds great promise.

#### 2.1.2 Non-iron chelators

In addition to iron chelators, several compounds have been found to inhibit ferroptosis by modulating iron metabolism and are used in disease treatment.

Yang et al. screened a non-classical inhibitor of ferroptosis, **YL-939**, from chemical libraries. They found that **YL-939** binds to its target inhibitor 2PHB2 and promotes ferritin expression to reduce iron levels to inhibit ferroptosis and alleviate ferroptosis-mediated liver injury (Yang et al., 2022). Compound **YL-939** provides a new intervention strategy for diseases associated with ferroptosis.

Phenotypic screening and structural modification identification of benzimidazole derivatives and discovery of a novel ferroptosis inhibitor compound **9a** by Fang et al. They found that compound **9a** inhibited ferroptosis mainly by stabilizing intracellular Fe^2+^. In the middle cerebral artery occlusion (MCAO) model, compound **9a** was found to significantly alleviate neurological impairment after IS. In addition, compound **9a** can bind to NCOA4 and break the NCOA4-FTH1 interaction (Fang et al., 2021).

A National Cancer Institute screen found that the polycyclic aromatic compound **NSC306711** blocked iron uptake by the Tf-TfR pathway and inhibited ferroptosis. At the same, unlike classical lattice protein-mediated endocytosis of Tf receptors, this drug is also known as ferritin due to its different previous endocytosis pathway that induces internalization and degradation of unoccupied Tf receptors (Horonchik and Wessling-Resnick, 2008).

Study demonstrates that DMT1 inhibitors reduce DMT1-mediated non-transferrin bound iron (NTBI) and play a role in ferroptosis-mediated disease (Sun et al., 2023). These include inhibitors such as **pyrrolidine dithiobarbamate (PDTC)** (Wetli et al., 2006) and **benzylisothiourea** (Zhang et al., 2012).

The complex Chinese herb **Naotai formula extract (NTE)** has been reported to modulate FPN-1, downregulate TFR1 and DMT1 levels, and reduce ROS and MDA accumulation (Yang T. et al., 2021). **NTE** was found to upregulate rat Recombinant Solute Carrier Family 7, Member 11 (SLC7A11), GPX4, and GSH levels in the MCAO model (Lan et al., 2020; Qiu et al., 2022). Meanwhile, the flavonoid compound **Carthamin yellow (CY)** has been shown in *ex vivo* and *in vivo* experiments to be useful in myocardial ischemia-reperfusion (MIRI) injury and to reduce ROS. In recent years, Guo et al. found downregulation of Fe^2+^, ROS, reverse transcription of Acyl-CoA Synthetase Long-Chain Family Member 4 (ACSL4), Fe^2+^, TFR1, Glutathione (GSH), SOD, and MDA levels in the brain and improvement of infarct size in the rat brain after administration of the drug in MCAO models (Guo et al., 2021; Chen G. et al., 2022). In addition, the natural flavonoid **Farrerol (FA)** may alleviate ferroptosis by inhibiting iron accumulation and lipid peroxidation (Wu et al., 2022).

### 2.2 Inhibition of ferroptosis via the lipid metabolism pathway

**FIGURE 2:**
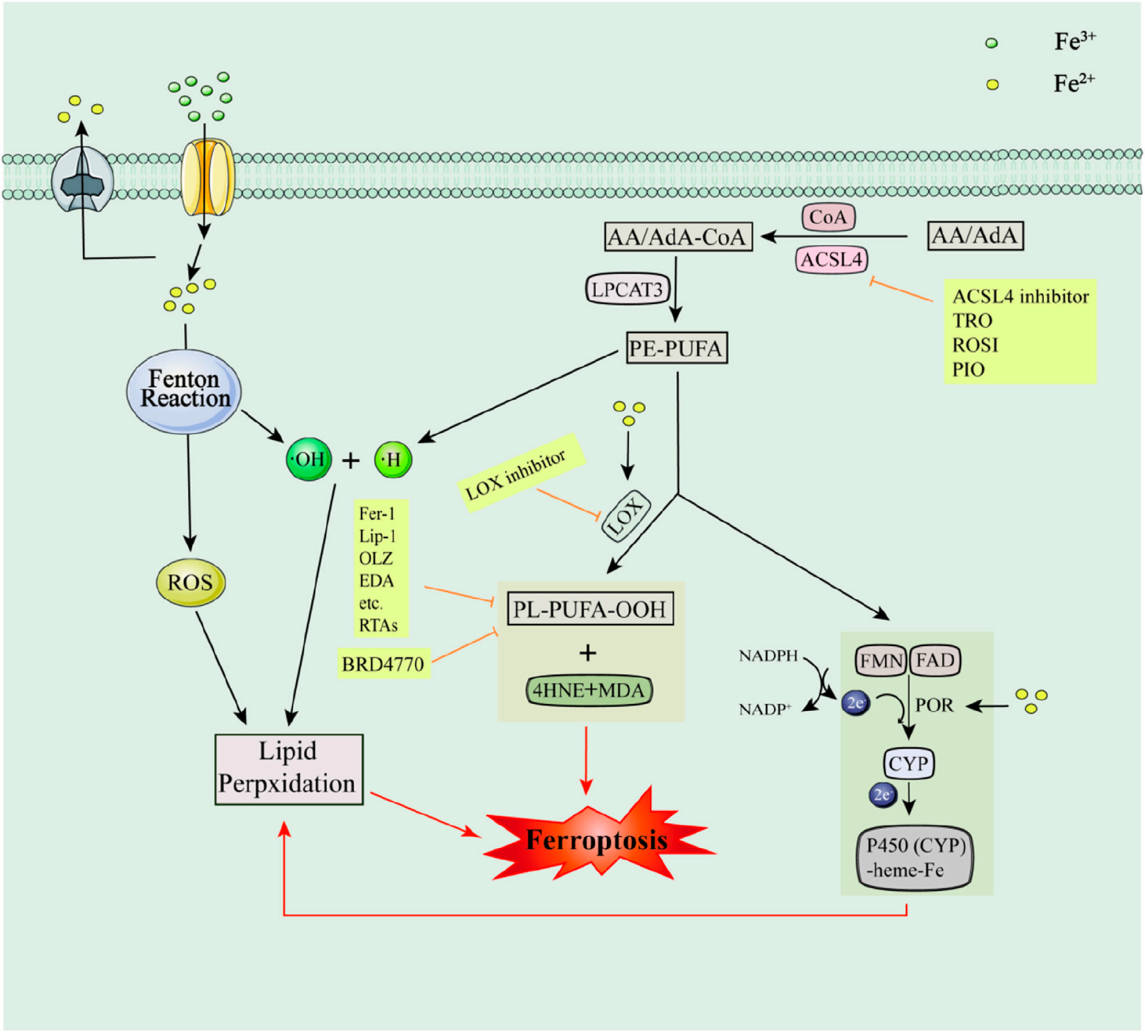
Lipid metabolic pathways and their associated inhibitors of ferroptosis. Acyl-CoA Synthetase Long-Chain Family Member 4 (ACSL4); Recombinant Solute Carrier Family 3, Member 2 (SLC3A2); Malondialdehyde (MDA); 4-hydroxynonenal (4HNE); Cytochrome P450 oxidoreductase (POR); Flavin mononucleotide (FMN); Flavin adenine dinucleotide (FAD).

Lipid peroxidation is central to triggering ferroptosis. It was shown that Polyunsaturated fatty acids (PUFA) of arachidonic acid (AA)/adrenaline (AdA) were acylated and esterified to AdA-Phosphatidyl Ethanolamine (AA-PE) and AA-PE with the participation of ACSL4, coenzyme A, and lysophosphatidylcholine acyltransferase-3 (LPCAT3). Ultimately, PE-PUFA leads to lipid peroxidation and induces ferroptosis through both non-enzymatic and enzymatic reactions (Naowarojna et al., 2023; Pope and Dixon, 2023). The non-enzymatic reaction that requires the participation of ROS generated by Fe^2+^ mediated Fenton reaction. In contrast, enzymatic reactions may be more complex. The enzymatic reaction generally catalyzes the oxidation of PUFAs with the involvement of LOX to produce PE-PUFA-OOH and its derivatives and reactive aldehydes, including Malondialdehyde (MDA) and 4-hydroxynonenal (4HNE), which in turn induce ferroptosis, but these aldehydes, in turn, lead to impaired nucleic acid and protein functions (Pope and Dixon, 2023). Cytochrome P450 oxidoreductase (POR) also promotes lipid peroxidation and induces ferroptosis with the involvement of two cofactors, Flavin mononucleotide (FMN) and Flavin adenine dinucleotide (FAD) (Koppula et al., 2021). In addition, iron can also catalyze the metabolic activity of two enzymes, LOX and POR (Zou et al., 2020) (e.g., Figure 2).

#### 2.2.1 Free radical trapping antioxidants

Free radical trapping antioxidants (RTAs) inhibit ferroptosis and protect hydrocarbon systems by trapping lipid peroxyl radicals. **Fer-1** and **Liproxstatin-1 (Lip-1)** (Zilka et al., 2017), both obtained by high-throughput screening of small molecule libraries, rapidly transfer H atoms from their arylamine portions to lipid radicals to inhibit lipid peroxidation and prevent free radical chain reactions in membrane PUFA lipids (Bayır et al., 2020; Scarpellini et al., 2023). Current researchers have identified quite a few other exogenous RTAs and many endogenous RTAs that play a role in iron-death-mediated diseases (e.g., Table 1).

**TABLE 1:** Small molecule ferroptosis inhibitors.

Sort	Inhibitors	Mechanism of action	Clinical diseases	Experimental models	Ref
Iron metab-olism	Deferoxamine (DFO)	Iron chelator (Fe^3+^); Upward revision of GPX4, FTH1, xCT	Myocardial ysfunction, Ischemia Reperfusion njury in the retina, Stroke, Spinal cord injury, etc.	SCI mice model, MCAO mice model	Yao et al. (2019), Zhang et al. (2020), Millán et al. (2021), Zeng et al. (2021), Guo et al. (2022b)
	Deferiprone (DFP)	Iron chelator	Multiple sclerosis, Beta-thalassaemia, Alzheimer’s disease, etc.	AD mice model, Beta-thalassaemia patients	Olivieri et al. (2019), Rayatpour et al. (2022)
	Deferasirox (DFX)	Iron chelator (Fe^3+^)	Cardiac iron overload, Ulcerative colitis, Beta-thalassaemia etc.	A mouse model with ulcerative colitis; Beta-thalassaemia patients	Olivieri et al. (2019), Wu et al. (2023)
	CN128	Iron chelator	Beta-thalassaemia, Parkinson’s disease	Thalassaemia beta-mouse model	Chen et al. (2020a)
	Dexrazoxane (DXZ)	Iron chelator; Upward revision of GPX4, FTH1	Cardiomyopathies	Rat model with cardiomyopathy; H9c2 cell model	Popelová et al. (2009), Zhang et al. (2021), Huang et al. (2024)
	Compound 9c	Iron chelator, Radical scavenger	Acute kidney injury	HT22 cell model; AKI model	Rayatpour et al. (2022)
	Ciclopirox (CPX)	Iron chelator	non-obese diabetic/severe combined immunodeficiency	Nonobese diabetic/severe combined immunodeficient murine xenograft model	Eberhard et al. (2009), Lin et al. (2021a), Lu et al. (2022)
	Baicalein	Iron chelator; Inhibit 12/15-LOX	Myocardial I/R Injury, Osteoarthritis, Acute kidney injury	Osteoarthritis mouse model; Mouse Myocardial I/R Injury model	Scarpellini et al. (2023)
	Hinokitiol	Iron chelator; Activate Nrf2; Upward revision of SLC7A11, GPX4, HO-1	Traumatic brain injury, Ischemic stroke, Parkinson’s disease	MCAO mice model; PD model	Jayakumar et al. (2013), Xi et al. (2022)
	Tannins	Iron chelator	Diseases related to iron overload	HepG2 cell model	Phiwchai et al. (2018)
	BMS536924	Iron chelator	—	—	Kuganesan et al. (2021)
	M-30	Iron chelator	Alzheimer’s disease	AD mice model	Avramovich-Tirosh et al. (2007), Zhang et al. (2022a)
	2,2′-Bipyridine	Iron chelator (Fe^2+^)	—	—	Chen et al. (2020b)
	1,10-Phenanth-roline	Iron chelator	—	—	Huang et al. (2019), Chen et al. (2020b)
	GIF-2197-r	Iron chelator	—	HT22 cell model	Hirata et al. (2023)
	Deferric Amine Compounds (DFA1)	Iron chelator	—	—	Feng et al. (2022)
	AKI-02	Iron chelator	Acute kidney injury	HK-2 cells model	Zhu et al. (2023b)
	YL-939	YL-939 binds to its target inhibitor 2PHB2 to promote ferritin expression and reduce iron content	Liver injury	ES-2 and HT-1080 cell model	Yang et al. (2022a)
	Compound 9a	Stabilisation of Fe^2+^	Ischemic stroke	HT22 cell model; HT1080 cell model	Fang et al. (2021)
	NSC306711	Induction of internalisation and degradation of unoccupied Tf receptors	—	—	Horonchik and Wessling-Resnick (2008)
	Pyrrolidine dithiobarbama-te	Inhibition of DMT1	—	HEK293T cell model	Wetli et al. (2006)
	Benzylisothiou-rea	Inhibition of DMT1	—	An acute rat model of iron overabsorption	Zhang et al. (2012)
	Benzimidazole compounds	Inhibition of NCOA4-FTH1 interaction	—	HT-22 cell model	Mallais et al. (2023)
	Carthamin yellow (CY)	Downward revision of Fe^2+^, ROS; Reverse transcription of ACSL4, Fe^2+^, TFR1, GSH, SOD, MDA expression	Myocardial I/R Injury, Ischemic stroke, etc.	MCAO rat model	Guo et al. (2021)
	Farrerol (FA)	Reduces lipid peroxidation and iron accumulation	Hypoxic-ischemic encephalopathy (HIE)	Tendinopathy rat model	Wu et al. (2022)
Lipid metabo-lism	Vitamin E	Radical scavenging; Reduction of Fe^3+^ to Fe^2+^	Seizures, Acute lung injury, Alzheimer’s disease, etc.	Chronic epilepsy rat model	Saito (2021), Zhang et al. (2022c), Tavakol and Seifalian (2022)
	Melatonin (MLT)	Radical scavenging; Activate Nrf2, HO-1; Downward revision of ROS	Myocardial injury, Ischemic stroke, Subarachnoid Hemorrhage, Retinal damage, Apoptotic Retinal Ganglion Cell Death, etc.	Mouse retinal ischemia-reperfusion injury model; Rat subarachnoid haemorrhage model	Kajarabille and Latunde-Dada (2019), Ma et al. (2023a), Zhang et al. (2023b)
	Vitamin K (VK)	Radical scavenging	Acute kidney injury	GPX4 gene deletion mice model; AKI mice model	Shearer and Okano (2018), Hirschhorn and Stockwell (2022), Kolbrink et al. (2022), Mishima et al. (2022), Li et al. (2023a)
	Ferrostatin-1 (Fer-1)	Radical scavenging	Cardiomyopathies, Stroke, Acute kidney injury, Acute Liver injury, etc.	HT-1080 cell model	Yang et al. (2021b), Scarpellini et al. (2023)
	Compound 37(SRS11-92)	Radical scavenging	—	HT-1080 cell model	Linkermann et al. (2014), Yang et al. (2021b)
	Compound 38 (UAMC-2418)	Radical scavenging	—	HT-1080 cell model	Linkermann et al. (2014), Yang et al. (2021b)
	Compound 39 (UAMC-3203)	Radical scavenging	—	HT-1080 cell model	Linkermann et al. (2014), Yang et al. (2021b)
	Liproxstatin-1 (Lip-1)	Radical scavenging	Myocardial I/R Injury, Acute kidney injury, Acute lung injury, Stroke	Renal tubular epithelium and hepatocyte-specific GPX4-deficient mice model	Alli et al. (2023)
	Compound 51	Radical scavenging	Ischemic stroke	MCAO rat model	Yang et al. (2021b)
	7J	Radical scavenging	—	HT-1080 cell model	You et al. (2022)
	XJB-5-131	Radical scavenging	Osteoarthritis, Renal I/R injury	HT-1080 cell model	Krainz et al. (2016), Charaschanya et al. (2022a)
	JP4-039	Radical scavenging	—	HT-1080 cell model	Krainz et al. (2016), Charaschanya et al. (2022a)
	(S)-6c	Radical scavenging	—	HT-1080 cell model; RAW 264.7 macrophage model	Charaschanya et al. (2022a), Charaschanya et al. (2022b)
	Copper complex diacetylbis (N (4)-methylthio- semicarbazonato) copper (II) (CuATSM)	Radical scavenging	Myocardial ischemic, Ischemic stroke, Amyotrophic lateral sclerosis	tMCAO model; ALS patient	(Kuo et al., 2019; Shi et al., 2021; Yang et al., 2023)
	CuATSP	Radical scavenging	—	PFA1 and HT-22 cells model	Zilka et al. (2021)
	Compound 25	Radical scavenging	—	HT-1080 cell model	Ying et al. (2024)
	Olanzapine (OLZ)	Radical scavenging	—	HT22 cell model	Jiang et al. (2023a)
	Phenoxazine	Radical scavenging	—	PFA1 cell model	Shah et al. (2017), Zilka et al. (2017), Farmer et al. (2022)
	Phenothiazine	Radical scavenging	—	PFA1 cell model	Yang et al. (2021b)
	Trolox	Radical scavenging	Cortical neuronal injury	HT-1080 cells model	Dixon et al. (2012)
	Pentamethylchromanol (PMC)	Radical scavenging	—	PFA1 cell model	Zilka et al. (2017), Shah et al. (2018b)
	SKI II (SphK-I2)	Sphingosine kinase (SphK) RTA	—	HT-1080 cells model	Conlon et al. (2021)
	Edaravone (EDA)	Activation of the Nrf2-FPN pathway predominantly; Upregulates Nrf2, FPN, GPX4, xCT; Downregulation of inflammatory factors, ACSL4 and LOX-5	Myocardial atrophy, Stroke, Spinal cord injury, Amyotrophic lateral sclerosis	CIRI, ICH and SCI mice model	Pang et al. (2022)
	BRD4770	Downregulates LPO and MDA; Upregulates SLC7A11, SLC3A2, GPX4, 4-HNE and FSP1	Aortic dissection	AD mice model	Chen et al. (2022b), Chen et al. (2022c)
	Zileuton	Inhibition of LOX-5; Attenuates lipid peroxidation; Blocking ALOX-5-mediated glutamate toxicity	Acute retinal demege	ARPE-19 cell model, Acute retinal injury mice model, HT22 cell mocel	Liu et al. (2015), Lee et al. (2022)
	PD146176	Selective inhibition of ALOX-15; Reduction of PLO and 4-HNE production	Alzheimer’s disease	Gamete mice model	Walters et al. (2018)
	MK-886	Inhibition of ALOX-5	Myocardial I/R Injury	Mouse Myocardial I/R Injury model	Shi et al. (2023)
	BWA4C	Inhibition of ALOX-5	Inflammatory bowel diseases	Rat bone remodelling model	Franchi-Miller and Saffar (1995)
	AA-861	Inhibition of ALOX-5/12	Ischemic stroke	Transient brain I/R gerbil model	Scarpellini et al. (2023)
	ML351	Specific inhibition of ALOX-15	Embolic stroke	A7r5 cell model, Murine Left Anterior Descending (LAD) Coronary Artery model	Cai et al. (2023a), Horinouchi et al. (2024)
	Troglitazone (TRO)	Inhibition of ACSL4	—	Rat model	Kim et al. (2001), Doll et al. (2017), Xiao et al. (2019)
	Rosiglitazone (ROSI)	Inhibition of ACSL4	—	Rat model	Kim et al. (2001), Doll et al. (2017), Xiao et al. (2019)
	Pioglitazone (PIO)	Inhibition of ACSL4	—	Rat model	Kim et al. (2001), Doll et al. (2017), Xiao et al. (2019)
	Silibinin	Inhibition of ACSL4	—	HepG2 cell model	Ghadi et al. (2023)
	PBSs7	Inhibition of ferroptosis	—	HT-1080 cell model	Li et al. (2023b)
	Gossypol acetic acid	Upregulates GPX4; Downregulates ACSL4 and Nrf2	Myocardial I/R injury, Osteoarthritis	Mouse Myocardial I/R Injury model	Lin et al. (2021b)
	Calycosin	Reduced accumulation of lipid peroxides	Diabetic nephropathy	HK-2 cell model, tubular damage mice model	Huang et al. (2022)
Antiox-idant	Metformin (Met)	Upregulates Nrf2/ARE, GPX4; Downregulates MDA	Spinal cord injury, Non-alcoholic fatty liver disease	SCI mice model	Wang et al. (2020b), Ma et al. (2021), Cai et al. (2023b)
	Paeoniflorin	Downregulates Fe^2+^, MDA, ROS, SLC7A11; Upregulates SOD	Cardiomyopathies, Alzheimer’s disease, Acute kidney injury	APP/PS1, AKImice model	Zhai et al. (2023a), Ma et al. (2023b)
	Carvacrol	Upregulates GPX4	Ischemic stroke	MCAO-I/R mice model	Abdul Ghani et al. (2023)
	Galangin	Upregulates SLC7A11, GPX4/Nrf2; Downregulates Iron, Ferritin	Cerebral I/R Injury, Myocardial I/R Injury	Cerebral I/R; MIRI mice model; Wistar rats model	Salama and Elshafey (2021)
	Ginkgolide B	Upregulates Nrf2/GPX4; Reversal of TFR1, NOCA4	Alzheimer’s disease	AD mice model	Wang et al. (2020a), Hébert et al. (2022)
	Kaempferol (KF)	Activation of the Nrf2/SLC7A11/GPX4 signalling pathway	Myocardial I/R Injury, Diabetic nephropathy, Ischemic stroke	OGD/R mice model	Holland et al. (2020), Kim et al. (2023)
	Glycyrrhizin	Adjustment of the HMGB1/GPX4 pathway; Upregulates GPX4	Myocardial I/R Injury, Cerebral I/R Injury	Neonatal hypoxic-ischemic brain damage (HIBD)	Jitrangsri et al. (2022), Zhu et al. (2022)
	Icaritin (ICT)	Upregulates GSH-Ps, SOD	Myocardial injury, Atherosclerosis, Ischemic stroke, Alzheimer’s disease	HIBD; OGD; APP/PS1mice model	Angeloni et al. (2019), Choi et al. (2023b), Zheng et al. (2023)
	Total flavonoids	Upregulates GSH; Downregulates ROS	Parkinson’s disease	PD mice model	Gao et al. (2024)
	Fisetin	Activation of the SIRT1/Nrf2 pathway; Promotion of FTH1 and HO-1 expression	Myocardial injury, Fibrosis Kidney Disease	Mouse Myocardial Injury model; H9c2 cell model	Goujon et al. (2024)
	Α-lipoic acid	Adjust the Xc-GSH-GPX4 axis; Downregulates ROS; Iron chelator	Myocardial Infarction, Acute kidney injury, Liver injury	AKI model	Cho et al. (2022)
	Berberine (BBR)	Upregulates GPX4; Downregulates Fe^2+^, ROS	Cardiomyopathies, Cardiotoxic, Cerebral I/R Injury, Liver fibrosis	pancreatic beta-cell	Manogaran et al. (2023)
	Gastrodin (GAS)	Regulating the GPX4 pathway	Cardiac hypertrophy, Kidney injury, Alzheimer’s disease	AKI mice model	Berezutsky et al. (2022)
	Ajudecunoid C	Interference with the Keap1-Nrf2 pathway and activation of the Nrf2-AREs pathway	Neuronal damage	Animal models of neurological diseases	Tan et al. (2021)
	Dehydroabietic Acid	Activation of the Nrf2-AREs pathway	Non-alcoholic fatty liver disease	NAFLD mice model	Gonçalves et al. (2018)
	Withaferin A	Activation of the Nrf2/HO-1pathway	Cerebral hemorrhage	ICH mice model	Zhou et al. (2023)
	Proanthocyanidins (PACs)	Upregulates GSH, GPX4, SLC7A11, Nrf2, HO-1; Downregulates TFR1, ACSL4; Activation of the Nrf2/HO-1pathway	Spinal cord injury, Cerebral I/R Injury, Acute lung injury	SCI, CIRI mice model	Zhou et al. (2020), Chen et al. (2023)
	Irisin	Activation of the Nrf2/HO-1pathway	Lung I/R injury	Lung I/R Injury mice model	Wang et al. (2022b)
	Aloe-emodin (AE)	Activate Nrf2; Upregulates SLC7A11, GPX4	Cardiac toxicity	H9c2 rat model	He et al. (2023b)
	Pachymic Acid	Downregulates MDA, ROS, Fe^2+^; Upregulates GSH, SLC7A11, GPX4	Myocardial injury, Ischemic stroke	OGD/R mice model; MI cell model	Liu et al. (2024)
	Geraniin	Upregulates Nrf2, HO-1	Kidney injury	MCAO/R and OGD/R model	Chumboatong et al. (2020)
	β-Caryophyllene	Activates the NRF2/HO-1 pathway; Downregulates ROS and iron accumulation	Myocardial infarction, Cardiac hypertrophy, Ischemic stroke	MCAO/R; ODG/R rat model	Hu et al. (2022a)
	Forsythoside A (FA)	Activates the Nrf2/GPX4 pathway; Upregulates GSH; Downregulates MDA, ROS	Alzheimer’s disease	APP/PS1 mice model; HT22 cell model	Zhang et al. (2024b)
	15, 16-Dihydrotanshinone I (DHT)	Upregulates GPX4 expression and GSH/GSSG ratio; Activate Nrf2; Downregulates ROS	Ischemic stroke	pMCAO rat model; PC12 cell model	Roth et al. (2023)
	Puerarin	Downregulates Fe^2+^, COX_2_; Upregulates Nrf2, SLC7A11, GPX4, HO-1	Myocardial injury, Retinal injury, Cerebral I/R Injury	OGD/R model	Yuan et al. (2017), Zhang et al. (2024a), Li and Liu (2024), Song et al. (2024)
	Eriodictyol	Upregulates Nrf2/HO-1; Downregulates ROS, MDA, Creatinine	Alzheimer’s disease	ALK mice model; APP/PS1 mice model	Li et al. (2022a), Zhang et al. (2022a), Badi et al. (2024)
	Dihydromyricetin	Regulation of Nrf2/HO-1, MAPK and NF-κB signalling pathways	Acute kidney injury, Cerebral I/R Injury	AKI mice model; HK-2cell model9C	Liang et al. (2014), Xu et al. (2023c)
	Naringenin	Adjustment of Nrf2/system Xc-/GPX4 axis, Nrf2-HO-1	Myocardial I/R Injury, Lung Fibrosis	MIRI rat model; H2 cell model	Shamsi et al. (2021), Zhang et al. (2022b)
	Hesperidin	Upregulates Nrf2	Cardiomyopathies, Parkinson’s disease	Intervertebral disc degeneration (IVDD) mice model	Zhu et al. (2023a)
	Nuciferine	Upregulates GPX4, SLC7A11 and FSP1; Downregulates iron	Acute kidney injury	AKI mice model; HK-2 and HEK293T cell model	Manogaran et al. (2023)
	(+)-Clausenamide	Activation of the Keap1/Nrf2 axis	Liver injury	Liver injury mice model	Wang et al. (2020c)
	Naringin	Regulation of the Nrf2/GPX4 pathway	Myocardial I/R Injury	Diabetic Mellitus rat model	Tang et al. (2022)
	Tectorigenin	Influence on NADPH oxidase 4 expression	Kidney injury	unilateral ureteral obstruction rat model	Li et al. (2022b)
	Biochanin A	Regulating the Nrf2/system Xc-/GPX4 pathway; Downregulates TFR1 and Ferritin levels	Osteoarthritis	Chondrocyte arthritis mouse model	He et al. (2023a)
	Isoiquiritin apioside	Upregulates HIF-α and HO-1	—	ALI mice model	Zhongyin et al. (2022)
	Polydatin	Downregulates iron, ROS; Upregulates GSH, GPX4	Acute kidney injury	Cis-AKI mice model; HK-2c ell model	Karami et al. (2022)
	Leonurine	Activate Nrf2; Downregulates iron, ROS; Upregulates GSH, GPX4	Myocardial injury, Acute kidney injury	AKI model	Hu et al. (2022b), Salama et al. (2022)
	Quercetin (QCT)	Activation of the Nrf2-HO-1 signalling pathway; Upregulates SLC7A11, SLC3A2, GSH; Downregulates MDA, ROS	Cardiomyopathies, Acute kidney injury, Chronic lung injury, etc.	AKI-I/R model	Cruz-Gregorio and Aranda-Rivera (2023), Feng et al. (2023), Kato et al. (2023), Ding et al. (2024)
	Resveratrol	Regulation of the Nrf2/GPX4 pathway; Regulation of SLC7A11/GPX4	Myocardial injury, Heart failure, Myocardial I/R Injury, Spinal cord injury, Ischemic stroke, etc.	SCI model	Li et al. (2023c), Huang et al. (2023), Ni et al. (2023)
	Arbtuin	Regulation of Nrf2/HO-1; Downregulates MDA, ROS; Upregulates GSH	Myocardial injury, Nalcoholic Fatty Liver Disease, etc.	NAFLD mice model; HepG2 cell model	Jiang et al. (2023c)
	3-n-butylphthalide	Downregulates iron, ROS, TFH; Upregulates Nrf2	Stroke, Alzheimer s disease, Ischemic diseases, Spinal cord injury	SH-SY5Y cell model	Ye et al. (2023)
	Tetrahydroxy stilbene glycoside (TSG)	Regulation of Nrf2/HO-1; Activation of GSH/GPX4/ROS and Keap1/Nrf2/ARE signalling pathways	Alzheimer’s disease	AD rat model; APP/PS1mice model	Pan et al. (2016); Zhao et al. (2023)
	Cardamonin	Regulation of the p53/SLC7A11/GPX4 signalling pathway	Osteochondral injury, Enterocolitis	Rat cartilage degeneration model	Gong et al. (2023)
	Ginsenoside Rg1	Downregulates iron, FTL, FTH and MDA; Upregulates GPX4, FSP1 and GSH	Kidney injury	Rats model of sepsis	Guo et al. (2022a), Guo et al. (2023a)
	Ruscogenin	Activation of the Keap1/Nrf2/HO-1 pathway	Myocardial ischemic, Acute kidney injury	MI mice model	Fu et al. (2022)
	Astaxanthin	Activates Nrf2, HO-1	Myocardial injury, Acute lung injury, Osteoarthritis, etc.	Neuroblastoma Human Cell Model	Rizzardi et al. (2022)
	2-amino-5-chloro-N,3-dimethylbenzamide (CDDO)	Inhibition of GPX4 degradation, lipid peroxidation; Downregulates ROS	Liver injury	HT-22 cell model	Wu et al. (2019)
	ADA-409–052	Inhibits tert-butyl hydroperoxide (TBHP)-induced lipid peroxidations; Prevention of ferroptosis due to GSH as well as GPX4 deficiency	Embolic stroke	A Murine Model of Thromboembolic Stroke	Keuters et al. (2021)
	Disulfiram	Disruption of GPX4 interaction with HSC70 and consequent inhibition of GPX4 degradation	—	mice model	Liu et al. (2022)
	Fursultiamine	Disruption of GPX4 interaction with HSC70 and consequent inhibition of GPX4 degradation	—	mice model	Liu et al. (2022)
	Mitoglitazone	Upregulates GPX4; Reduction of lipid peroxidation and alleviation of AKI in mice after I/R	Renal I/R injury	renal I/R injury mice model	Qi et al. (2023)
	PKUMDL-LC-101	Activation of GPX4 to reduce the production of pro-inflammatory lipid mediators	—	human polymorphonuclear leucocytes	Li et al. (2018), Li et al. (2019), Scarpellini et al. (2023)
	PKUMDL-LC-101-D04	Activation of GPX4 to reduce the production of pro-inflammatory lipid mediators	—	human polymorphonuclear leucocytes	Li et al. (2018), Li et al. (2019), Scarpellini et al. (2023)
	Dopamine (DA)	Enhanced stability of GPX4 protein; Upregulates GPX4, FTH1; Downregulates ROS	Myocardial I/R Injury, Parkinson’s disease, Ischemic stroke	PD model; MCAO model	Costa and Schoenbaum (2022), Ding et al. (2023)
	Seratrodast	Regulation of the Xc - GSH-GPX4 pathway; Enhanced GPX4 expression; Downregulates ROS	Seizures	Seizures in Mouse Models	Noack et al. (2017)
	Uridine	Activation of the Nrf2 signalling pathway SLC7A11, GPX4 and HO-1; Activation of the Nrf2 signalling pathway	Acute kidney injury	ALI model	Lai et al. (2023)
	3H-1, 2-dithiole-3-thione (D3T)	Upregulates xCT, GSH, Nrf2-mediated ferritin and FPN1, HO-1	Alzheimer’s disease	AD mice model	Cui et al. (2018), Kulkarni et al. (2023)
	5- amino-3-thioxo 6- 3H-(1,2) dithiole-4-carboxylic acid ethyl ester (ACDT)	Upregulates xCT, GSH, Nrf2-mediated ferritin and FPN1, HO-1	Alzheimer’s disease	AD mice model	Ansari et al. (2018)
	Propofol	Adjustment Nrf2/GPX4 Access	Myocardial I/R Injury, Cerebral I/R Injury	CIRI mice model	Fan et al. (2023)
	Compound 3f	Upregulates FSP1	Ischemic stroke	Rat MCAO model	Fang et al. (2022)

##### 2.2.1.1 Endogenous free radical trapping antioxidants

**Vitamin E** and the trace mineral selenium (**Se**) form a complementary antioxidant system (Saito, 2021). **Vitamin E** inhibits lipid peroxide production by reducing Fe^3+^ in LOX-15 (Tavakol and Seifalian, 2022), but it acts less potently than **Fer-1** and **Lip-1**. Furthermore, it was found that neurological damage caused by **vitamin E** deficiency in COVID-19 patients was strongly associated with ferroptosis. (Tavakol and Seifalian, 2022).

**Melatonin (MLT) is different from vitamin E** (Zhang et al., 2023). It can regulate ferritin, lipid peroxidation, and antioxidant capacity (Kajarabille and Latunde-Dada, 2019). **MLT** was found to inhibit ferroptosis in subarachnoid haemorrhage-mediated neuronal injury by activating genes such as Nrf2 and Heme oxygenase-1 (HO-1) (Ma et al., 2023). Furthermore, **MLT** alleviates retinal damage and retinal ganglion cells (RGC) death by inhibiting p53-mediated ferroptosis (Zhang et al., 2023). Thus MTL is expected to be a potential therapeutic agent for the treatment of ferroptosis-mediated diseases.

**Vitamin K (VK)** is a redox-active naphthoquinone, including chlorophyll quinone, menaquinone-4 (MK-4), and menaquinone in three forms (Hirschhorn and Stockwell, 2022). **VK** is converted to hydroquinone (VKH2) by **VK** epoxide reductase (VKOR) (Shearer and Okano, 2018). And in a mouse model with a genetic deletion of GPX4, MK-4 showed a protective effect on tissues (Mishima et al., 2022). At the same time, **VK** reductase Ferroptosis Inhibitory Protein 1 (FSP1) inhibits ferroptosis by reducing **VK** to VKH2 and prevents lipid peroxidation by depleting NAD(P)H (Li et al., 2023). In addition, recently Kolbrink et al. reported that **VK1** may act as a potent endogenous antioxidant to ameliorate AKI (Kolbrink et al., 2022).

##### 2.2.1.2 Exogenous free radical trapping antioxidants

**Fer-1**, the first synthetic inhibitor of ferroptosis, stabilises free radicals, reduces ROS, and has been strongly implicated in a variety of diseases (Skouta et al., 2014; Wang et al., 2023), including acute lung injury (ALI) (Liu et al., 2020). In structure-activity relationship (SAR) analysis (Saito, 2021), it was found that N-cyclohexyl acts as a lipophilic anchor playing an important role in maintaining **Fer-1** activity (Scarpellini et al., 2023). Moreover, both amine groups and lipophilic anchors are essential for maintaining **Fer-1** activity. Studies have reported that **Fer-1** inhibits ferroptosis both *in vivo* and *in vitro* with significant *in vitro* inhibition. Structural analysis of **Fer-1** by researchers yielded the structurally stable compound **SRS11-92**, but it is less active and less stable in plasma metabolism (Linkermann et al., 2014). Further researchers used elastin-induced HT-1080 cells as a model of ferroptosis, using **SRS11-92** as a starting point for optimization. They first introduced a sulfonamide instead of the unstable ester and introduced a benzyl ring 2 on the NH to obtain the sulfonamide analogue compound **38 (UAMC-2418)**. Compound **38** has high stability but low solubility. Thus, the researchers introduced solubility-enhancing groups into **compound 38** to obtain compound **39 (UAMC-3203)**. Compound **39** showed stronger solubility, stability, and pharmacokinetic values than **38**, was protective against multiple organ damage in mice showed no toxicity, and was overall superior to **Fer-1** (Scarpellini et al., 2023). Meanwhile, **the nanomaterial poly(2-oxazoline)-Fer-1** significantly improved the potency of **Fer-1** (Morrow et al., 2022), providing a new idea for the treatment of ferroptosis.

**Lip-1** is a newly discovered ferroptosis inhibitor that contains a spiroquinoxaline amine scaffold, functioning similarly to **Fer-1** without affecting other cell death pathways. It is both active and soluble, and was introduced around the same time as the **Fer-1** derivative **UAMC-3203**. Various research studies have highlighted the significance of **Lip-1** in treating different neurological disorders and cancers. A recent study revealed that a **Lip-1** analog, **Lip-2**, successfully prevented ferroptosis induced by serum in human proximal tubular epithelial cells from patients with lupus nephritis (Class IV), while also exhibiting improved pharmacokinetics (Alli et al., 2023).

To obtain more potent inhibitors, Yang et al. screened the phenothiazine derivatives isoprozine as well as phenothiazine from the Selleck (USA) library of biologically active compounds and they further explored the structure–activity of phenothiazine derivatives (Shah et al., 2017). By substituting different functional groups, they found that compound **51** with methylcytosine had better activity and exerted its antioxidant capacity mainly by trapping free radicals, thus protecting against elastin-induced cellular ferroptosis. It is worth noting that the compound has good pharmacokinetics as well as good BBB penetration, which is essential for the treatment of CNS diseases. Compound **51** was further evaluated in the MCAO model, which showed a significant reduction in lesion volume (Yang et al., 2021). However, it has a high hERG activity, so they further optimized the structure based on the antioxidant and ferroptosis inhibition properties of compounds with phenothiazine scaffolds to obtain 2-vinyl-10H-phenothiazine derivatives. Compound **7J** was found to have the best ferroptosis inhibitory activity by SAR study, **7J** showed good ROS scavenging ability and could alleviate DOX-induced cardiotoxicity with a good pharmacokinetic profile and no significant toxicity *in vitro* and *ex vivo* (You et al., 2022).

**Edaravone (EDA)**, a free radical scavenger approved for treating ischemic stroke (IS) and amyotrophic lateral sclerosis (ALS) (Rothstein, 2017). Recent studies have reported that **EDA** predominantly activates the Nrf2-FPN pathway, upregulates Nrf2, FPN, and GPX4, and downregulates inflammatory factors to inhibit ferroptosis and alleviate cerebral ischemia-reperfusion injury (CIRI). In addition, it was found that **EDA** upregulated GPX4 and xCT, downregulated ACSL4 and LOX-5, and inhibited neuronal cell ferroptosis during the acute phase of SCI (Pang et al., 2022).

A novel ferroptosis inhibitor, **olanzapine (OLZ)**, was found to trap free radicals and inhibit ferroptosis in RSL3-induced hippocampal neuronal cells of HT22 mice (EC_50_ = 1.18 μM) (Jiang et al., 2023; Drabczyk et al., 2023). To improve its inhibitory effect, the researchers optimized its structure. Forty-two thiophene benzodiazepine derivatives (compounds **4–45**) were first designed and synthesized and seven compounds (compound **21** and compounds **31–36**) were selected by analyzing their tectonic relationships for better ferroptosis inhibition activity (Jiang et al., 2023). After HT22 cytotoxicity evaluation compound **36** was found to have low cytotoxicity (CC_50_ = 18.8 μM) and its inhibitory activity was 16 times higher than that of **OLZ** (EC_50_ = 0.074 μM). Thus, the discovery of **OLZ** derivatives solves the problem of easy metabolism and low efficacy of **Fer-1** and offers hope for ferroptosis-mediated diseases (Jiang et al., 2023).

Recent studies have reported that mitochondria-targeted nitrogen oxide RTA has an effective inhibitory effect on ferroptosis. Nitrogen oxides catalyze the cross-disproportionation of alkyl peroxyl and hydroperoxyl radicals, allowing them to form two substances in unsaturated hydrocarbons as uniquely effective RTAs. Ability of Targeted Nitrogen Oxides **XJB-5-131** and **JP4-039** to prevent the Occurrence of Cytosolic ferroptosis in HT-1080 Cells as Olefinic Peptide Isoforms (Krainz et al., 2016). Optimisation of **JP4-039** by Manwika Charaschanya et al. found **compound (S)-6c** to be the most potent inhibitor of ferroptosis in HT-1080 cells (approximately 30 times more active than **JP4-039**) (Charaschanya et al., 2022a; Charaschanya et al., 2022b).

In recent years, copper complex diacetylbis **(N(4)-methylthio- semicarbazonato) copper(II) (CuATSM)** (Kuo et al., 2019) has been an efficient RTA. The study reports that **CuATSM** reduces the area of murine cerebral infarction and oxidative stress and exerts antioxidant and neuroprotective effects in acute IS in a transient MCAO (tMCAO) model (Shi et al., 2021). **CuATSM** can be used in combination with **EDA** to upregulate this effect (Shi et al., 2021). In addition, **CuATSM** also exerts a neuroprotective effect in ALS, but the pathological mechanism is currently unknown (Kuo et al., 2019; Yang et al., 2023).

The investigators phenotypically analyzed a new 4-hydroxyl pyrazole scaffold for ferroptosis inhibitor **4-hydroxyl pyrazole derivatives (HW-3)** (EC_50_ = 120.1 ± 3.5 nM) and synthesized a series of 4-hydroxyl pyrazole derivatives based on the backbone structure of **HW-3**. And it was found that compound **25** exhibited the strongest inhibition of ferroptosis (EC_50_ = 8.6 ± 2.2 nM). In addition, cellular-level studies have found that compound **25** exhibits more potent inhibition of ferroptosis than **Fer-1** (Ying et al., 2024), offering hope for ferroptosis-mediated diseases.

#### 2.2.2 Lipoxygenase inhibitors

Lipoxygenase (LOX) is thought to be a central player in ferroptosis. It induces lipid peroxidation by reacting with ROS and catalysing PUFA, which in turn induces ferroptosis. It has been found that humans have six LOX isoforms, ALOX5, ALOX12, ALOX12B, ALOX15, ALOX15B, and ALOXE3, and there are already some cells that can be rescued by LOX inhibitors. LOX inhibitors inhibit LOX primarily by trapping free radicals (Shah et al., 2018a), which in turn inhibits lipid peroxidation. The most relevant inhibitors reported in the article are LOX-5 inhibitors, including **zileuton** (Liu et al., 2015; Lee et al., 2022), **MK-886** (Shi et al., 2023), **BWA4C** (Franchi-Miller and Saffar, 1995), **PD146176** (Walters et al., 2018) and others. Furthermore, it was found that the LOX-5 inhibitor **Zileuton** is involved in oxidative stress in retinal pigment epithelium (RPE) cells and regulates retinal ROS. It provides an effective solution for the treatment of retinal diseases (Liu et al., 2015; Lee et al., 2022). LOX-5 expressed during inflammation uptakes apoptotic cells, activates resident macrophage populations, and thus maintains apoptotic cell tolerance (Kapralov et al., 2020). Also, **ML351** specifically inhibits ALOX-15 and alleviates erastin-induced cardiac ischemia/reperfusion (I/R) injury (Cai et al., 2023; Horinouchi et al., 2024). In addition, the LOX-5/12 inhibitor **docebenone (AA-861)**, and the LOX-12/15 inhibitor **baicalein** have all been found to inhibit lipid peroxidation (Scarpellini et al., 2023) and play a role in ferroptosis-mediated diseases.

#### 2.2.3 Acyl-CoA Synthetase Long-Chain Family Member 4 inhibitors

The insulin sensitising drugs thiazolidinediones (TZD) are a class of peroxisome proliferator activatedreceptor γ (PPARγ) activators. **Troglitazone (TRO)**, **rosiglitazone (ROSI)** and **pioglitazone (PIO)** were found to effectively and specifically inhibit ACSL4 (Kim et al., 2001), and **TRO** being TZD-protective due to its 6-chromophoranol structure (Doll et al., 2017). Pharmacological evaluation of ACSL4 revealed that GPX4KO mice treated with **ROSI** showed better inhibition of ferroptosis (Doll et al., 2017). In addition, **thrombin inhibitors** also inhibit ACLS4 and ferroptosis (Zhang et al., 2024a).

#### 2.2.4 Other inhibitors

**Histone methyltransferase inhibitors (BRD4770)** is comparable to **Fer-1** inhibition at optimal concentrations (Chen et al., 2022b; Chen et al., 2022c) and may be useful in the treatment of stenosis. The researchers screened and found that **BRD4770** exhibited significant ferroptosis inhibition. They further found that **BRD4770** inhibited lipid peroxidation by down-regulating PLO and MDA levels and increasing 4-HNE expression. And **BRD4770** upregulated the mRNA levels of ferroptosis regulators SLC7A11, Recombinant Solute Carrier Family 3, Member 2 (SLC3A2), GPX4, and FSP1. Furthermore, it was found that **BRD4770** alleviated cognitive impairment and downregulated aortic lipid peroxidation in a mouse model of AD (Chen et al., 2022b). Thus, **BRD4770** is expected to be an effective molecular drug for the treatment of AD.

Recently **Florencio Porto Freitas et al. identified 7-dehydrocholesterol (7-DHC)**, an endogenous ferroptosis inhibitor, as one of the lipid components susceptible to autotrophication *in vivo*. They prepared soybean phosphatidylcholine (PC) monolayers loaded with **7-DHC** and found that **7-DHC** is preferentially oxidised *in vitro* and that the oxidation of **7-DHC** *in vitro* is critical for the inhibition of (phospho)lipid peroxidation. Subsequently, using the iron/ascorbate couple as a model for oxidative sources, they found that **7-DHC** accumulation protects (phosphoric acid) lipids from autoxidation and subsequent rupture and is different from previous lipid peroxidation, mainly due to its better reactivity to peroxyl radicals. In addition, **7-DHC** accumulation significantly reduces metabolic stress in the body (Angeli et al., 2021; Freitas et al., 2024). Thus, **7-DHC** regulates ferroptosis in an easily overlooked manner by inhibiting lipid peroxidation and bringing new therapeutic ideas to ferroptosis-mediated diseases.

In recent years, researchers have isolated seven abolane-type sesquiterpenoids (PBSs) from the deep sea fungus Aspergillus floridus YPH1 (Li et al., 2023b), with compound **7** showing a selective inhibitory effect on Erastin/RSL3-induced ferroptosis similar to **Fer-1**. However, unlike **Fer-1**, compound **7** exhibited negligible radical scavenging activity in 2,2-diphenyl-1-picrylhydrazyl (DPPH) assays (Li et al., 2023b). Additionally, **PBSs25 gossypol acetic acid (GAA)** was found to protect cardiomyocytes from ferroptosis *in vitro* by reducing chelated iron and lipid peroxidation. *In vivo* studies showed that **GAA** significantly upregulated GPX4 (Lin et al., 2021).

### 2.3 Inhibition of ferroptosis via antioxidant action

**FIGURE 3:**
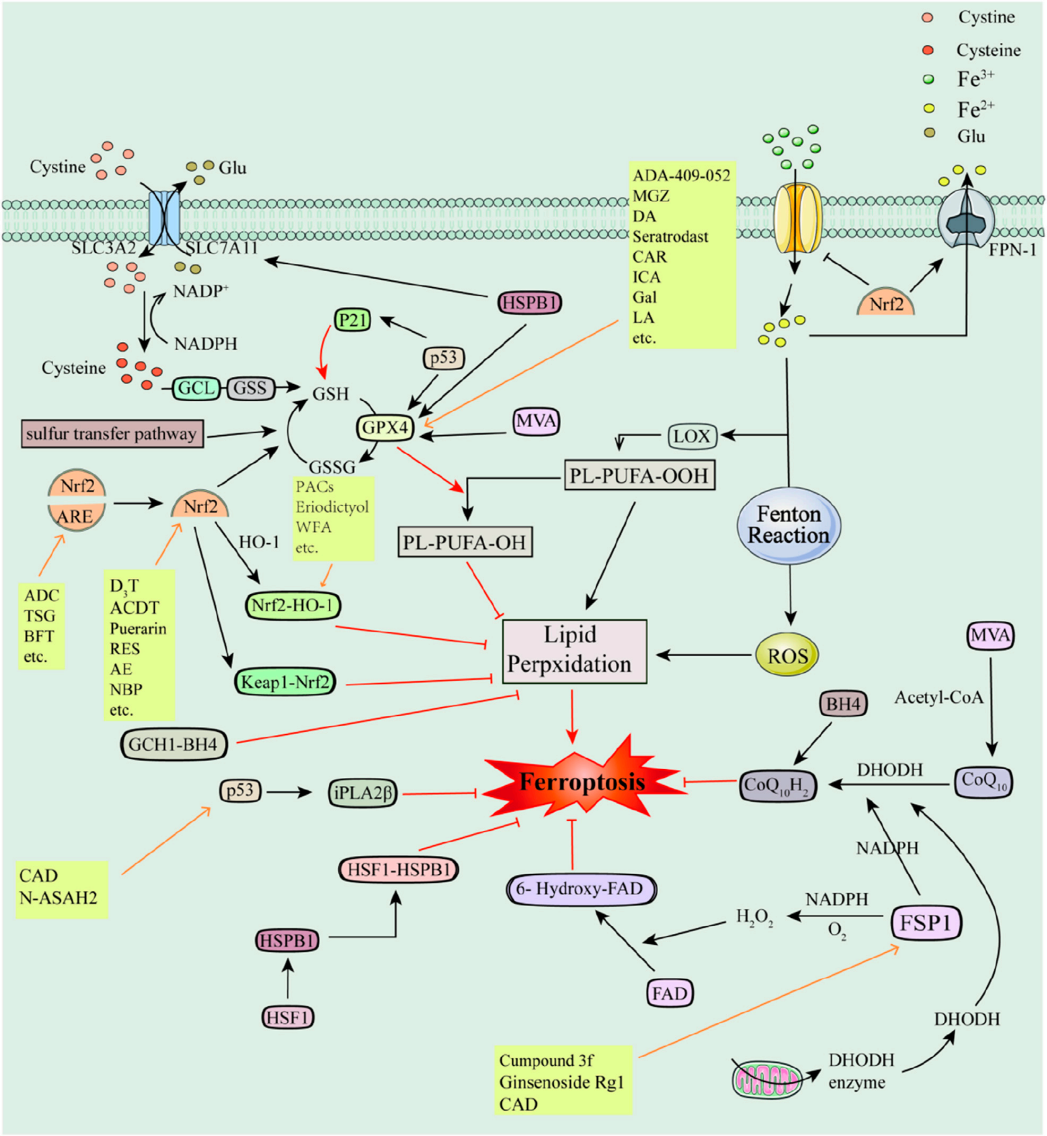
Antioxidant pathways and their associated inhibitors of ferroptosis Recombinant Solute Carrier Family 7, Member 11 (SLC7A11); Recombinant Solute Carrier Family 3, Member 2 (SLC3A2); Glutamate-cysteine ligase (GCL); Glutathione Synthase (GSS); Glutathione (GSH); Glutathione Peroxidase 4 (GPX4); Oxidized glutathione, GSSG; Glutamic acid (Glu); Cystine (Cys); Nuclear factor E2-related factor 2 (Nrf2); p53 (tumor suppressor); Recombinant Kelch-like ECH Associated Protein 1 (Keap1); Antioxidant response element (ARE); Heme oxygenase-1 (HO-1); Ferroptosis Inhibitory Protein 1 (FSP1); Coenzyme Q_10_ (CoQ_10_); Panthenol (CoQ_10_H_2_); Nicotinamide Adenine Dinucleotide Phosphate (NADPH); Dihydroorotate dehydrogenase (DHODH); GTP Cyclohydrolase-1 (GCH1); Tetrahydrobiopterin (BH4); GTP Cyclohydrolase-1-Tetrahydrobiopterin (GCH1-BH4); Mevalonate (MVA); Heat Shock Protein Beta-1 (HSPB1); Heat shock factor (HSF1); 6-hydroxy-FAD; p53-iPLA2β axis; p21; Acetyl-coenzyme A (Acetyl-CoA).

The antioxidant system is central to impeding ferroptosis by stabilizing or extinguishing free radicals and thereby inhibiting lipid peroxidation (Tan et al., 2023) (e.g., Figure 3.). System Xc-is an important antioxidant system involved in the regulation of ferroptosis. System Xc-passes through a heterodimer of the light-chain subunit SLC7A11 and the heavy-chain subunit SLC3A2, and glutamate-cysteine ligase (GCL) and glutathione synthase (GSS) transfer cystine to the cell and synthesise glutathione (GSH). Then, GSH is converted to Oxidized glutathione (GSSG) with the participation of glutathione peroxidation GPX4 to eliminate toxic lipid peroxides (Dar et al., 2024). Moreover, in the presence of GSH GPX4 converts toxic lipid peroxides into non-toxic lipocalciferol, which protects cells from lipid peroxidation and thus inhibits ferroptosis (Dar et al., 2024).

The anti-oxidative stress transcription factor Nrf2 and the tumor protein p53 are the two most studied transcription factors in ferroptosis. Nrf2 regulates a variety of antioxidant enzymes and proteins to exert antioxidant effects and inhibit ferroptosis, including Superoxide Dismutases (SODs), HO-1, NAD(P)H-associated enzymes, GPX4, catalase (CAT), FPN, TFR, TF, System Xc-, ferrochelatase (FECH), etc. (Punziano et al., 2024). p53, as an effective oncogene, promotes ferroptosis mainly through the canonical and non-canonical pathways (Dixon et al., 2012). However, recent studies have found that the p53-GPX4 axis eliminates lipid peroxidation, the p53-iPLA2β axis eliminates free radicals, and p53 transactivates iPLA2β and inhibits ferroptosis when cells are low in damage from lipid peroxidation (Gnanapradeepan et al., 2022). In addition, p53 induces GSH production by P21 (also known as cell cycle protein-dependent kinase inhibitor 1A, CDKN1A), which in turn upregulates GPX4 thereby inhibiting ferroptosis (Tarangelo and Dixon, 2018; Tarangelo et al., 2018; Tarangelo et al., 2022) (e.g., Figure 3).

FSP1 can inhibit ferroptosis by generating H_2_O_2_ in the presence of O_2_ and NADPH and then converting FAD to 6-hydroxy-FAD (Lv et al., 2023; Nakamura et al., 2023). Meanwhile, FSP1 can reduce Coenzyme Q_10_ (CoQ_10_) to panthenol (CoQ_10_H_2_) with the involvement of NADPH and thus inhibit ferroptosis (Li et al., 2023; Zhang et al., 2023). CoQ_10_ is a lipophilic RTA (Li et al., 2023; Zhang et al., 2023). Furthermore, the NADPH-FSP1-CoQ_10_ pathway is independent of the Xc-GSH-GPX4 pathway and cooperates with it to inhibit lipid peroxidation and ferroptosis (Bersuker et al., 2019). Dihydroorotate dehydrogenase (DHODH) can also independently reduce CoQ_10_ to CoQ_10_H_2_, thereby protecting cells from lipid peroxidation (Amos et al., 2023a; Amos et al., 2023b) (e.g., Figure 3).

Other antioxidant pathways involved in ferroptosis include GTP Cyclohydrolase-1-Tetrahydrobiopterin (GCH1-BH4), Heat shock factor-Heat Shock Protein Beta-1 (HSF1-HSPB1), the mevalonate (MVA) pathway, Sulfur transfer pathway, the glutaminolysis pathway, and so on. Overexpression of the ferroptosis regulator GCH1 inhibits lipid peroxidation and promotes BH4 synthesis (Xu et al., 2023). BH4 is a potent endogenous RTA and participates in CoQ_10_H_2_ formation. The GCH1-BH4 pathway has endogenous antioxidant effects and is independent of the Xc-GSH-GPX4 axis and the NADPH-FSP1-CoQ_10_ axis (Akiyama et al., 2023). Transcription factor HSF1 promotes HSPB1 transcription and forms the HSF1-HSPB1 pathway (Sun et al., 2015). HSPB1 is a negative regulator of ferroptosis that upregulates GPX4, SLC7A11, and G6PD and HSPB1 overexpression attenuates ischemic-hypoxic brain damage in neonatal rats (Doshi et al., 2010; Liang et al., 2023). MVA plays a regulatory role in ferroptosis. On the one hand, by regulating selenocysteine tRNA which in turn promotes GPX4 synthesis. And on the other hand, MVA can synthesize CoQ_10_ in the presence of acetyl coenzyme A, which in turn participates in the Xc-GSH-GPX4 axis and the NADPH-FSP1-CoQ_10_ pathway and is involved in the regulation of ferroptosis (Xing et al., 2023). The Sulfur transfer pathway plays a role in the maintenance of redox homeostasis and oxidative stress, mainly due to the upregulation of intracellular Cys, GSH, and GSSG and the inhibition of ROS as a result of Cys-tRNA synthetase (CARS) deficiency (Floros et al., 2022; Sun et al., 2023). Glutamine catabolism provides sufficient GSH and ATP for ferroptosis and also plays a supporting role in ferroptosis-mediated diseases (Durante, 2019; Shin et al., 2020; Gagliardi et al., 2023; Xiao et al., 2023) (e.g., Figure 3). Several ferroptosis inhibitors have been reported to inhibit ferroptosis by modulating the antioxidant pathway.

#### 2.3.1 The System Xc-GSH-GPX4 axis

The heat shock protein 90 (HSP90) plays an important role in protein maturation, stabilisation, and activation (Wickramaratne et al., 2023). A triterpenoid **compound 2-amino-5-chloro-N, 3-dimethylbenzamide (CDDO)** was identified by Wu et al. It inhibits HSP90, which in turn inhibits GPX4 degradation and lipid peroxidation, and downregulates ROS, protecting cells from damage caused byferroptosis (Wu et al., 2019). Furthermore, in 2022 Liu et al. found that a series of disulfide compounds, such as **disulfiram (DSF)** and **fursultiamine**, could disrupt the interaction of GPX4 with HSC70, which in turn inhibited GPX4 degradation and protected cells from ferroptosis (Liu et al., 2022).

In 2021 Meike Hedwig Keuters et al. found that the novel ferroptosis inhibitor **arylthiazyne derivative small molecule (ADA-409-052)** inhibited tert-butyl hydroperoxide (TBHP)-induced lipid peroxidation and prevented ferroptosis induced by GSH, as well as GPX4 deficiency. It is rapidly absorbed after oral administration in a mouse model. In addition, administration of the drug in a thrombotic mouse model revealed a significant decrease in the area of cerebral edema, the area of cerebral infarction, and the expression of pro-inflammatory factors in mice (Keuters et al., 2021). Thus, small molecule inhibitors such as **ADA-409-052** offer new therapeutic strategies for neurological disorders as well as acute brain injury.

In 2023, Qi et al., 2023 reported that the antidiabetic drug **mitoglitazone (MGZ)** upregulated GPX4 and reduced lipid peroxidation, thereby significantly alleviating renal injury in mice after I/R, providing a new therapeutic strategy to ameliorate renal I/R injury. In addition, **MGZ** can also exert a nephroprotective effect by maintaining the normal morphology of mitochondria.

The catecholamine neurotransmitter **Dopamine (DA)** plays a role in human cognitive functions. It was shown that **DA** enhances the stability of GPX4 protein (Costa and Schoenbaum, 2022). Increased survival of **DA** ergic neurons and reversal of ROS, GPX4, and FTH1 levels in SNpc after moxibustion application in a Parkinson’s disease (PD) model and improvement of motor deficits in it. And **DA** agonists reduce the risk of side effects during recovery (Ding et al., 2023). In addition, **levodopa** is approved for use in patients with early or late stroke and can be used in combination with physiotherapy (Obi et al., 2018). **DA**-mediated ferroptosis has been mentioned in other diseases and is expected to lead to new research directions in ferroptosis-mediated diseases.

The study reports that Se inhibits ferroptosis and protects neurons by enhancing GPX4 expression. **Se** acts in the antioxidant pathway mainly by containing selenoproteins (Ramakrishnan et al., 2022; Choi et al., 2023). The study reports that **Se** inhibits ferroptosis and protects neurons by enhancing GPX4 expression (Alim et al., 2019). Inadequate levels of organismal **Se** downregulate GPX4 and antioxidant enzymes and upregulate ROS, MDA, and LPO (Xu et al., 2023). **Se** alleviates cerebral ischemia-induced neurological damage by activating GPX4 in IS (Alim et al., 2019). Iron, MDA and 4-HNE were found to be downregulated and promote the FSP1/GPX4 pathway after sodium selenite injection in a rat model of SCI, which in turn improved motor function in rats (Chen et al., 2022). In addition, injection of double selenium nanospheres **(CLNDSe)** in an AD mouse model revealed abnormal microglia protofibrils or tau protein-targeted aggregation outside of Aβ42, and its crossing of the BBB into the brain significantly downregulated ROS, ACSL4, and COX-2, and upregulated FTH1, GPX1, and GPX4, ultimately inhibiting ferroptosis and ameliorating cognitive deficits in APP/PS1 mice (Solovyev et al., 2018). Overall, the trace element **Se** has been found to play a role in a variety of diseases by mediating ferroptosis.

Recently Li et al., 2019 identified a potent GPX4 metastable site and obtained eight GPX4 metastable activators by structural analysis and computational design (Scarpellini et al., 2023). They inhibit ferroptosis by activating GPX4 enzyme activity (IC_50_ > 100 μM). **Compound PKUMDL-LC-101** and its analogue **PKUMDL-LC-101-D04** most efficiently activated and increased GPX4 activity in intact cells while inhibiting ferroptosis in a cellular model (Scarpellini et al., 2023). These compounds provide an effective strategy for the future development of activators for other protein targets and also lead to new therapeutic strategies for lipid peroxidation-related diseases such as neurodegenerative diseases (Li et al., 2018; Li et al., 2019).

Upregulation of GPX4 levels and downregulation of lipid ROS in neurons after treatment with the thromboxane A2 receptor antagonist **Seratrodast** and consequent inhibition of neuronal cell ferroptosis in erastin-induced hippocampal HT22 cells. And **Seratrodast** increased GPX4 expression and shortened seizure duration and prolonged seizure latency in a mouse model of epilepsy (Noack et al., 2017). Thus **Seratrodast** could play a role in several diseases by inhibiting ferroptosis.

Using APP/PS1 mice as subjects, researchers found that the monoterpene glycoside **paeoniflorin (PF)** improved cognitive performance and downregulated the levels of Fe^2+^, MAD, and ROS in brain tissue, which reduced oxidative damage and thus alleviated the neurological damage in AD mice (Zhang and Wei, 2020; Zhai et al., 2023). And **PF** can reverse AKI by inhibiting SLC7A11-mediated ferroptosis (Ma et al., 2023). In 2019, Guan et al. found that the monoterpene oenol **carvacrol (CAR)** (Abdul Ghani et al., 2023) reduced PLO levels in ischemic gerbil brain tissue and inhibited ferroptosis by up-regulating GPX4, which exerted neuroprotective effects in both *in vivo* and *ex vivo* models of IS. Thus, **CAR** has the potential to be an effective therapeutic agent for IS. In 2021, Li et al. found that injection of **Ginkgolide B (GB)**, the active ingredient of terpene lactones (Wang et al., 2020), in an animal model of AD reversed the levels of TFR1 and NOCA4 and upregulated the expression of Nrf2 and GPX4 in the brains of SAMP8 mice, which exhibited neuroprotective effects in AD mice (Hébert et al., 2022).

The lanolin-type triterpenoid **Pachymic Acid (PA)** has a variety of pharmacological properties. Liu et al. established a cellular myocardial infarction (MI) model after administration of 20 μg/mL and found that the inhibitory effect of oxygen-glucose deprivation/reperfusion (OGD/R) on cell viability was reversed and the levels of MDA, ROS, and Fe^2+^ were downregulated, and the expression of GSH, SLC7A11, and GPX4 was increased. Meanwhile, **PA** inhibits cardiomyocyte ferroptosis in a dose-dependent manner and attenuates MIRI injury in mice (Liu et al., 2024).

Flavonoids are an important class of phenolic metabolites in plants with good antioxidant effects. The bioflavonoid **Kaempferol (KF)** has shown neuroprotective effects in neurological disorders such as IS and AD. Recently, it was found that **KF** activated the Nrf2/SLC7A11/GPX4 signalling pathway and enhanced antioxidant capacity, which in turn reversed OGDR-induced ferroptosis (Holland et al., 2020; Kim et al., 2023) and alleviated neuronal cell damage. The natural flavonoid compound **Icaritin (ICA)** plays a role in a variety of diseases such as IS, AD, depression, and others. It directly binds to Nrf2 and promotes GPX4 transcription, which in turn inhibits ferroptosis after IRI and ameliorates brain damage (Choi et al., 2023). In recent years, **ICA** compounds significantly enhanced GSH-Ps, SOD activities and alleviated oxidative stress injury in mouse brain tissue after gastric administration of **ICA** compounds in APP/PS1 double transgenic mice. In addition, **ICA** was found to alleviate cognitive deficits in AD mice in a dose-dependent manner in the Morris water maze (VWM) (Angeloni et al., 2019; Zheng et al., 2023).

**Total flavonoids from Aspergillus membranaceus (TFA)** play a role in neurodegenerative diseases. Gao et al., 2024 found that **TFA** prevented SH-SY5Y cell neurotoxicity by increasing GSH and GSH/GSSG ratios and decreasing ROS, and showed significant neuroprotection in MPTP/MPP-induced *in vitro* and *in vivo* PD mouse models. Simultaneous injection of the flavonoid **galangin (Gal)** after I/R injury in VWM revealed a significant reduction in lipid peroxidation levels and an upregulation of SLC7A11 and GPX4 expression in the gerbil brain, which resulted in the inhibition of ferroptosis and the protection of hippocampal neurons in the gerbil brain after I/R (Guan et al., 2021; Hassanein et al., 2023). At the same time, gerbils showed significant improvement in the area of learning memory. Recently, Yang et al. found that **Gal** prevented iron overload and lipid peroxidation in a MIRI model and significantly attenuated myocardial fiber damage, reduced cerebral infarct size, and improved cardiac function by targeting the GPX4/Nrf2 signalling pathway in mice after MIRI (Salama and Elshafey, 2021). Thus flavonoids are a promising inhibitor of ferroptosis.

**Α-lipoic acid (LA)** reverses folic acid (FA)-induced AKI. In 2021, Xue et al. found that **LA** upregulated GSH, GPX4, and downregulated ROS as well as lipid peroxidation, and that supplementation with **LA** reversed the low expression of SLC7A11 (Li et al., 2021). In addition, **LA** also inhibits ferroptosis and attenuates fluoride-induced liver injury by modulating the Xc-GSH-GPX4 axis and chelating iron (Cho et al., 2022).

In addition to the above-mentioned small molecule drugs, many herbal medicines have also been found to play a role in herbal medicines have also been found to play a role in ferroptosis-mediated diseases. For example, Lv et al. established a diabetic nephropathy (DN) mouse model and found that Fe^2+^ was downregulated and SLC7A11, GPX4 content, and GSH/GSSG content, and GSH/GSSG were upregulated in the renal tissues of mice after **San-Huang-Yi-Shen capsule (SHYS)** administration and that it alleviated renal injury (Su et al., 2021; Lv et al., 2023). At the same time, **Modified Shoutai Pill**, also known as **Jianwei Shoutai Pill (JSP)**, upregulated GSH, GPX4, downregulated MDA levels, and ACSL4 protein expression in the placenta of Recurrent Pregnancy Loss (RPL) mice, and was shown to protect against RSL3-induced lipid metabolism (Zhang et al., 2023; Lai et al., 2024). Futhermore, **Angong Niuhuang Wan (AGNHW)** has recently been found to exert neuroprotective effects by modulating GPX4-related signaling pathways and inhibiting ferroptosis (Bai et al., 2024).

#### 2.3.2 Nrf2

**Uridine**, composed of uracil and ribose, exhibits anti-inflammatory (Jeengar et al., 2017), antioxidant (Adant et al., 2022), and anti-aging (Krylova et al., 2021) properties. Kai Lai et al. (2023) discovered an increase in uridine phosphorylase 1 (UPP1) in an ALI model induced by lipopolysaccharide (LPS), leading to elevated uridine levels and the upregulation of SLC7A11, GPX4, and HO-1 expression. Moreover, Uridine was found to inhibit macrophage ferroptosis by activating the Nrf2 signaling pathway, with the protective effect enhanced by **Fer-1**. Therefore, supplementing with Uridine may offer potential therapeutic benefits for ALI (Lai et al., 2023).

Dithiolethiones, lipophilic organosulfur compounds (Ansari et al., 2018), such as **3H-1,2-dithiole-3-thione (D3T)** and **5-amino-3-thioxo-3H-(1,2)dithiole -4-carboxylic acid ethyl ester (ACDT)**, activate Nrf2 and increase system Xc- and GSH levels in the erastin-induced ferroptosis model, demonstrating antioxidant effects and significant inhibition of ferroptosis akin to **Fer-1** (Kulkarni et al., 2023). Treatment with **D3T** and **ACDT** upregulated Nrf2-mediated expression of ferritin and FPN1, while also protecting U-87MG cells from iron overload-induced cytotoxicity (Kulkarni et al., 2023). Additionally, **D3T** upregulates silent information regulator 1 (SIRT1), Nrf2, and HO-1, ameliorating cognitive deficits in the Tg2576 AD mouse model (Cui et al., 2018). Notably, **D3T** exhibits neuroprotective effects in neuroinflammation and IS, suggesting its potential as a treatment option for ferroptosis-related diseases.

In 2022, Fan et al., 2023 discovered that the anaesthetic **Propofol** (Islam et al., 2020) could have a strong antioxidant effect by modulating Nrf2/GPX4 through protein blotting, transmission electron microscopy, and glutathione assays. Furthermore, in a mouse model of CIRI, **Propofol** was shown to effectively prevent ferroptosis and protect against neuronal damage in the brain after cerebral ischemia/reperfusion.

**Metformin (Met)**, a biguanide derivative commonly used in the first-line treatment of type 2 diabetes, has also shown promise in various other conditions such as cancer, cardiovascular disease, and neurological disorders (Ala and Ala, 2021). Recent research has unveiled Met’s potential role in spinal cord injury (SCI) by inhibiting oxidative stress through the Nrf2/ARE pathway (Wang et al., 2020) and promoting neural regeneration post-SCI, offering new therapeutic possibilities. Additionally, studies have indicated that **Met** upregulates GPX4 (Ma et al., 2021), reduces MDA levels, and provides protection against neurological impairments following cerebral ischemia (Cai Z. et al., 2023).

The novel chlorane diterpenoid analogue **Ajudecunoid C (ADC)** is more effective in ferroptosis inhibition. It has been shown that **ADC** mainly acts on the Nrf2-AREs pathway and scavenges free radicals to exert an ferroptosis inhibitory effect, and **ADC** may interfere with the Keap1-Nrf2 pathway and activate the Nrf2-AREs pathway (Tan et al., 2021). In addition, the diterpenoid Dehydroabietic Acid activates the Keap1/Nrf2-ARE signalling pathway and attenuates non-alcoholic fatty liver disease (NAFLD). Therefore, researchers have proposed that diterpenoids may be effective compounds for inhibiting ferroptosis (Tan et al., 2021).

Biopolyphenolics **Proanthocyanidins (PACs)** are effective free radical scavengers. **PACs** have been reported to also have the ability to regulate LOX (Zhou et al., 2020). **PACs** have been found to inhibit ferroptosis and play a role in a variety of diseases by altering the expression of iron-related factors. For example, **PACs** upregulate the expression of GSH, GPX4, SLC7A11, Nrf2, and HO-1 and downregulate the expression of TFR1, ACSL4 and regulate SCI, thereby promoting ferroptosis-mediated functional recovery in SCI mice (Zhou et al., 2020). In addition, a large number of studies have shown that **PACs** activate the Nrf2-HO-1 pathway and ameliorate CIRI, bringing a new direction for CIRI treatment (Chumboatong et al., 2020). In 2022, Yuan et al. found downregulation of oxidative stress and neuronal death after injection of the phenolic substance **geraniin** by the middle cerebral artery occlusion-reperfusion (MCAO/R), and OGD/R models, as well as by neurological scoring assays, CCK8 assessment, TUNEL staining, detection of cells by flow cytometry, and Western blotting assessment (Chen et al., 2021). Meanwhile, they found that the infarcted portion of brain tissue upregulated *ex vivo* and *in vivo* Nrf2, HO-1 protein expression in a concentration-dependent manner in the tMCAO model (Chumboatong et al., 2020). Thus, the neuroprotective effect of **geraniin** may be closely related to the Nrf2/HO-1 signalling pathway, but further experimental elucidation is needed in the future.

Flavonoids have been implicated in ferroptosis-related diseases through their interaction with Nrf2. For example, **quercetin (QCT)**, a natural flavonoid, has been shown to alleviate renal injury by activating the Nrf2-HO-1 signaling pathway (Kato et al., 2023). Studies using an AKI-I/R model demonstrated that **QCT** administration led to increased cell viability, upregulation of ALC7A11, SLC3A2, and GSH expression, as well as a reduction in MDA and lipid ROS content in mice (Cruz-Gregorio and Aranda-Rivera, 2023). **QCT** also shows promise in the treatment of neurological disorders such as Parkinson’s disease (PD) (Jiang et al., 2023). **Eriodictyol**, another flavonoid, was found to upregulate Nrf2/HO-1 and reduce ROS levels in a renal injury model, providing protection against CP-induced AKI (Badi et al., 2024). Furthermore, **Eriodictyol** has been shown to inhibit ferroptosis and improve cognitive deficits in APP/PS1 mice through activation of the Nrf2/HO-1 pathway (Li et al., 2022; Zhang et al., 2022). **Naringenin**, a flavonoid derivative from citrus extract, has demonstrated efficacy in alleviating myocardial injury by modulating the Nrf2/system Xc-/GPX4 axis (Shamsi et al., 2021). It also shows potential in mitigating ferroptosis induced by silver nanoparticles in human bronchial epithelial BEAS-2B cells through the Nrf2/HO-1 axis (Zhang et al., 2022b). **Hesperidin** and **naringin** have both been found to inhibit ferroptosis by upregulating the Nrf2 pathway, offering protection to human myeloid cells (Tang et al., 2022; Zhu et al., 2023).

The isoflavone **tectorigenin** protects against unilateral ureteral obstruction and renal injury in rats by affecting NADPH oxidase 4 (NOX4) expression (Li et al., 2022). **Biochanin A**, an active isoflavone of Astragalus membranaceus, inhibits ferroptosis by modulating the Nrf2/system Xc-/GPX4 signalling pathway, decreasing TFR1 and ferritin levels, and relieves knee osteoarthritis (KOA) (He et al., 2023). Licorice extract chalcone **isoiquiritin apioside** inhibits ferroptosis mediated ALI through upregulation of HIF-α and HO-1 proteins (Zhongyin et al., 2022). **Puerarin**, a natural isoflavone extracted from Pueraria Mirifica, was found to be protective against neuronal cell damage in mice by modulating Nrf2 in the OGD/R model (Li and Liu, 2024). It was also demonstrated to downregulate Fe^2+^, cyclooxygenase 2 (COX2), and upregulate Nrf2 and its downstream related ferritin (including SLC7A11, GPX4, HO-1) expression to reduce lipid peroxidation and inhibit retinal ferroptosis (Song et al., 2024). In addition, **Puerarin** was found to downregulate p53 and effectively improve neurological impairments in patients with ischemic brain injury in combination with conventional treatment in the clinic (Hou et al., 2024).

The non-flavonoid polyphenolic compound **Resveratrol (RES)** was found to promote ferroptosis-mediated recovery of motor function in SCI mice by modulating the Nrf2/GPX4 pathway in an SCI model (Ni et al., 2023). Meanwhile, **RES** protects human bronchial epithelial cells (BEAS-2B) from ferroptosis by acting on the ferroptosis pathway and inhibits diabetic periodontitis-induced ferroptosis in alveolar osteoblasts (Li et al., 2023c), which offers the possibility of disease treatment. Furthermore, Kosuke Kato et al. showed that **quercetin** and **RES** inhibit ferroptosis by inhibiting iron-catalyzed hydroxyl radicals and are independent of the Nrf2-ARE pathway (Kato et al., 2023).

The natural molecule **Hinokitiol** has a stronger iron chelating ability than **DFO**. Platycodone activates Nrf2, upregulates SLC7A11, GPX4, heme oxygenase-1 (HO-1) and exerts antioxidant effects (Abeydeera et al., 2022; Xi et al., 2022). The study demonstrated that the injection of flatulin in the rat MCAO model significantly inhibited ferroptosis and reduced the size of cerebral infarcts and alleviated the neurological damage after cerebral ischemia in rats, providing new insights into the IS (Jayakumar et al., 2013). Meanwhile, **Hinokitiol** alleviates neurological aspects of behavioural disorders caused by the neurotoxin 6-hydroxy DA (6-OHDA), providing a new treatment option for PD (Xi et al., 2022). Thus **Hinokitiol** holds promise for more diseases.

The natural anthraquinone derivative **aloe-emodin (AE)** attenuates adriamycin (the doxorubicin, DOX)-induced cardiomyocyte toxicity in H9c2 rats (He Y. et al., 2023). The researchers assessed the molecular mechanism of action of Nrf2 by Western blot, luciferase reporter gene assay, and qRT-PCR analysis, and detected the changes of intracellular ROS, and lipids by fluorescence assay. The results showed that **AE** could activate Nrf2, upregulate the expression of SLC7A11 and GPX4, and exhibit significant antioxidant capacity, thus reducing oxidative stress (He et al., 2023).

The bioactive steroid ester **Withaferin A (WFA)** modulates endothelial cell apoptosis after traumatic brain injury (Şeker Karatoprak et al., 2022). In recent years, **WFA** has been experimentally found to have the ability to inhibit ferroptosis and to play a role in many diseases associated with ferroptosis. It has been shown that **WFA** can activate the Nrf2/HO-1 pathway and reduce oxidative stress to inhibit neuronal cell injury after ICH, thus exerting neuroprotective effects. Meanwhile, the combination of **WFA** with **Fer-1** increased this neuroprotective effect (Zhou et al., 2023), bringing new hope for ICH treatment.

**Arbutin (ARB)** was found to reduce ROS, and MAD accumulation, upregulate GSH, and exert a mitigating effect on alcoholic fatty liver disease (ALD) and liver injury by modulating the Nrf2/HO-1 pathway and oxidative stress. In addition, **ARB** can promote m6A methylation of SLC7A11 by inhibiting FTO, which in turn inhibits ferroptosis and exerts a mitigating effect on NAFLD *in vitro* and *in vivo* (Jiang et al., 2023).

**Dl-3-n-butylphthalide (NBP)**, an extract of celery, has been widely used in stroke, dementia, and ischemic diseases. In 2022 Ye et al. suggested that injection of **NBP** inhibited erastin-induced accumulation of iron and ROS, downregulated TFH, and upregulated Nrf2, which in turn inhibited ferroptosis and reversed the damage to DAergic neurons (MES23.5 cells) (Ye et al., 2023).

**Tetrahydroxy stilbene glycoside (TSG)**, an active ingredient of Polygonum tigrinum, enhances memory and locomotor activity in aged rats mainly by restoring mitochondrial function and plays a role in AD (Gao et al., 2023). In an AD rat model, **TSG** administration modulates the Nrf2-HO-1 pathway and protects against hippocampal neuronal damage in mice. Also **TSG** enhanced antioxidant capacity by activating GSH/GPX4/ROS and Keap1/Nrf2/ARE signalling pathways (Zhao et al., 2023). Recent studies have shown that **BFT**, a fat-soluble derivative of vitamin B1 synthesis, can also rescue cognitive deficits in mice in the APP/PS1 mouse model through activation of the Nrf2/ARE pathway (Pan et al., 2016).

In addition to the above drugs, small molecule drugs such as **Dihydromyricetin (DHM)** (Liang et al., 2014; Xu et al., 2023), **β-Caryophyllene(BCP)** (Hu et al., 2022), **Forsythoside A (FA)** (Zhang et al., 2024b), **15, 16-Dihydrotanshinone I (DHT)** (Roth et al., 2023) (Table 1) have been demonstrated to play a role in neurological impairment-related disorders by modulating the signalling factors associated with the Nrf2 pathway. However, their specific mechanisms still need to be verified more further.

#### 2.3.3 Other antioxidant pathways

Phenotypic analysis of the HT22 cell model revealed that the diphenylbutene derivative **DPT** could inhibit ferroptosis (EC_50_ = 12.0 mM) and was non-toxic. Researchers have designed and synthesised 14 **DPT** analogues (Fang et al., 2022) using **DPT** as a backbone and chemically to enhance the inhibitory activity. By analysis, it was found that **3f, 3m, and 3n** exhibited stronger inhibition of ferroptosis. Next, scaffolding analysis was carried out and it was found that R1 of **compound 3f** was substituted with 3-OCH3 and 4-OH on the A ring, and R1 of **compounds 3m** and **3n** was substituted with 3-OCH3 and 4-OH on both the A and B rings. A comparison of activities revealed that **compound 3f** was significantly more active than the latter two mentioned above. Also, the stronger ferroptosis inhibition ability and superior biological activity of **compound 3f** were demonstrated by morphological analysis. Further DPPH assay **DPT** was found to be different from RTAs such as **Fer-1** and **DFO** but inhibited ferroptosis by up-regulating FSP1 protein levels (Fang et al., 2022). In addition, **compound 3f** was found to alleviate the impairment of neurological function after cerebral ischemia to a certain extent in a rat MCAO model (Fang et al., 2022), which is expected to be a new prospect for the treatment of neurological disorders.

**Ginsenoside Rg1**, an active component of ginseng, has been shown to protect the kidney from damage by reducing oxidative stress. Following **Rg1** treatment, iron content, FTL, FTH, and MDA levels in renal tissues were significantly reduced, while GPX4, FSP1, and GSH levels were elevated (Guo et al., 2022). Recent studies have demonstrated that knocking down FSP1 eliminates the inhibitory effect of **ginsenosides** on ferroptosis, and the administration of **ginsenosides** effectively alleviates Sepsis-induced Acute Kidney Injury (SI-AKI) (Guo et al., 2023).

*In vivo* experiments have shown that the natural chalcone **cardamonin (CAD)** inhibits ferroptosis and improves cartilage damage in rats by modulating the p53/SLC7A11/GPX4 signaling pathway. Additionally, **CAD** has been found to have a similar effect as **DFO** in osteoarthritis (OA), with **CAD** being more effective in improving cartilage damage in OA (Gong et al., 2023).

## 3 Alternative approaches to targeting ferroptosis

In addition to the small molecule ferroptosis inhibitors traditionally used to inhibit ferroptosis that can play a role in ferroptosis-mediated diseases, gene regulatory approaches, cellular therapies, and nano-targeted delivery can also exhibit ferroptosis inhibition by all along the correlates of ferroptosis (Table 2), and there are some differences in treatment efficiency between them (Table 3).

**TABLE 2:** Alternative approaches to targeted inhibition of ferroptosis.

Methods of control		Mechanism of action	Ref
Gene regulation method	Reversal of BPD by miR-134-5p inhibitors	Inhibition of ROS, Fe^2+^ accumulation; Upregulates GPX4	Lan et al. (2023)
	MiR-3587 inhibitor protects renal tissue from IR injury	Upregulates HO-1, GPX4 expression and cellular activity	Tao et al. (2021)
	LncRNA-N1LR protects against brain damage after IS-I/R	Inhibits p53 phosphorylation and inactivates p53	Wu et al. (2017)
	Inhibition of ferroptosis by lncRNA-SNHG14	Downregulation of miR-206, upregulation of SLC7A11 expression	Li et al. (2023d)
	LncRNA-MEG3 alleviates ferroptosis in chondrocytes	Regulation of miR-885-5p-SLC7A11 signalling pathway and upregulation of GPX4	Zhu et al. (2024a)
	Prominin2 inhibits ferroptosis	Transporting iron out of the cell	Yan et al. (2022)
	PII inhibitor K-181 mitigates IS	Inhibition of p53 transcription	Yan et al. (2022)
	Silencing of ELAVL1 alleviates ischemic Brain damage in rats	Upregulates GSH, GPX4, SLC7A11 expression and cell viability and reduces Fe^2+^, ROS, MAD levels	Du et al. (2022)
	SEC24B	Alteration of iron regulatory proteins or transferrin to maintain iron homeostasis	Ryan et al. (2023)
	CDGSH iron-sulfur structural domain 2 (CISD2)	Activation of the Nrf2/HO-1 signalling pathway and downregulation of Fe^2+^	Hu et al. (2023)
Cell therapy	Neural stem cells transplanted to obtain NSCs-NRG1β	Upregulation of GPX4, SLC7A11 levels and downregulation of p53 expression	(Chen et al., 2021c; Zhai et al., 2022; Zhai et al., 2023b)
	“ neutrophil piggybacking” strategy	Neutrophil therapy in combination with Se targets delivery of antioxidant enzymes and upregulates GPX4	(Wang et al., 2022a; Xu et al., 2024)

**TABLE 3:** Comparing the advantages and disadvantages of different ferroptosis inhibition methods.

Sorts		Advantages	Disadvantages	Ref
Small molecule inhibitors	DFO			Tartaglione et al. (2020)
	DFX		1. Short half-life	
	Fer-1	1. Lower cost and easy to carry	2. Poor patient adherence	
	Lip-1etc.	2. High oral availability	3. Structural instability	
Gene regulation method	MiR-134-5p, MiR-3587, etc.	1. Expression stabilization and less likely to be damaged	1. Off-target effects	Akbari Moqadam et al. (2013)
		2. Associated with the pathological process of malignant tumors	2. Selectiveness	
		3. High detection accuracy		
	LncRNA-N1LR, LncRNA-SNHG14, etc.	1. Carrying more information, more diverse and more specific	1. Low exon levels	Chowdhury et al. (2024)
		2. High correlation with neighboring genes	2. Low level of expression	
		3. Obstruction of miRNA function	3. Subject to epigenetic influences	
Cell therapy	Neural stem cells transplanted to obtain NSCs-NRG1β etc.	1. Time-saving, simpler testing procedures	1. High costs, separation and purification operations are difficult	Park et al. (2024)
		2. Long-lasting and sustaining action	2. Easily and abnormally differentiated themselves	
		3. Can grow and transform to replace damaged cells	3. Uncertainty	
		4. Good organizational adaptation to the body		
Nanoparticles	Pulmonary drug-delivery system (PDDS), PCC-R8-ROS@miR-134-5p, SOD-PLGA-NPs etc.	1. Well-targeted	1. Limited knowledge of interactions between nanomaterials and human cells	Naskar and Kim (2019)
		2. Prolongation of drug Circular time or half-life	2. Difficulty in controlling the toxicity of nanomaterials	
		3. Easily penetrating biobarriers		
		4. Less side effects		

### 3.1 Gene regulation method

MicroRNAs (miRNAs) are small non-coding RNAs that negatively regulate gene expression and are implicated in various diseases. In a study by Huang et al., in 2022, miR-134-5p was found to be upregulated in bronchopulmonary dysplasia (BPD) in preterm infants, leading to ROS and Fe^2+^ accumulation and GPX4 downregulation in these patients. Conversely, inhibiting miR-134-5p reversed these effects and significantly improved BPD (Lan et al., 2023). Another key miRNA, miR-3587, regulates HO-1 and is involved in renal ischemia-reperfusion injury. Tao et al. demonstrated in 2022 that injecting a miR-3587 inhibitor post-establishment of an *ex vivo* IR model enhanced HO-1 and GPX4 expression and cellular activity, thus safeguarding renal tissues from IR injury (Tao et al., 2021).

lncRNAs are long-stranded non-coding RNAs, more than 200 nt in length, which can affect downstream miRNAs but cannot be converted into proteins. In 2017, Wu et al. found that lncRNA-N1LR was neuroprotective in IS-I/R by inhibiting p53 phosphorylation on serine 15 and inactivating p53 (Wu et al., 2017). lncRNA-SNHG14 was significantly upregulated in the nutlin3a-resistant osteosarcoma (OS) cell line NR-SJSA1, which resulted in downregulation of miR-206, upregulation of SLC7A11 expression and inhibition of ferroptosis (Li et al., 2023). lncRNA-MEG3 overexpression modulates miR-885-5p-SLC7A11 signalling pathway, upregulates GPX4, and thereby alleviates erastin-induced ferroptosis in chondrocytes (Zhu et al., 2024).

Prominin2, a pentameric transmembrane protein, plays a crucial role in mediating iron efflux. It facilitates the formation of multivesicular bodies (MVBs) and exosomes containing iron-rich proteins, allowing the removal of excess intracellular iron via these exosomes, thus preventing cellular ferroptosis. Additionally, Fer-1 has been shown to mitigate the decline in cellular function resulting from prominin2 depletion (Brown et al., 2019). In a study conducted in 2017, Yan et al., 2022 discovered that the small molecule protein-protein interaction (PPI) inhibitor K-181 could suppress p53 transcription and ameliorate neurological impairments post-ischemic stroke (IS) by increasing the expression of the p53 repressor Mdmx, consequently inhibiting p53 transcription in the brains of mice following IS. Embryonic lethal-abnormal vision like protein 1 (ELAVL1) is an RNA-binding protein that enhances mRNA stability and regulates translation, thereby influencing gene expression (Du et al., 2022). ELAVL1 is notably upregulated in the I/R model. Research has shown that silencing ELAVL1 leads to an increase in GSH, GPX4, and SLC7A11 expression, while decreasing Fe2+, ROS, and MAD levels in rat brain tissues, ultimately preventing ferroptosis and reducing ischemic brain damage in rats (Du et al., 2022).

In addition, several other genes have been discovered to influence ferroptosis-related diseases by regulating factors associated with ferroptosis. For instance, in 2022, Sean K. Ryan et al. identified the ferroptosis susceptibility gene SEC24B through a comprehensive gene screening process. This gene inhibits ferroptosis and is implicated in neurological disorders by modulating iron-regulated proteins such as transferrin, thus maintaining iron homeostasis within cellular iron pools (Ryan et al., 2023). In a separate study in 2023, Hu et al., 2023 demonstrated that CDGSH iron-sulfur domain 2 (CISD2) was upregulated and activated the Nrf2/HO-1 signaling pathway in both the MCAO mouse model and the OGD/R HT22 cell model, mimicking *in vivo* and *ex vivo* conditions of cerebral ischemia and reperfusion. This activation led to increased survival rates of HT22 cells, while also reducing Fe^2+^ content and exhibiting antioxidant properties. Furthermore, CISD2 was found to be effective in conditions such as IS-I/R and ICH (Yeh et al., 2022).

### 3.2 Cellular therapy

#### 3.2.1 Neuregulin1β

With the development of stem cell research, the use of neural stem cells (NSCs) in the treatment of diseases is now of great interest. It has been found that exogenous NSCs can repair damaged tissues and play a role in neurological diseases such as IS (Calabrese et al., 2022). In 2022, Zhai et al. introduced human umbilical cord mesenchymal stem cells (hUC-MSCs) obtained by growth factor induction into NSCs and added NSCs by adding growth factor and neuregulin1β (NRG1β) to obtain NSCs-NRG1β. They found improved neurological function and reduced area of cerebral infarction in the rats injected with NSCs-10 nM NRG1β group in the rat MCAO/R model, and they also found an increase in the levels of GPX4 and SLC7A11 and a downregulation of p53 expression (Zhai et al., 2022). 2023, they obtained the same results with NSCs-10 nM NRG1β intervention in oxygen OGD/R-injured PC12 cells (Zhai et al., 2023). Thus NSCs provide a new direction for the future treatment of neurological diseases. However, many issues including ethical issues, therapeutic efficacy, and safety of exogenous NSC transplantation remain to be resolved.

#### 3.2.2 Neutrophils therapy

To address the problems of antioxidant enzymes in IS therapy, in 2022, Wang et al. designed an albumin-conditioned nanoparticle based on co-encapsulation with antioxidases catalase (CAT) and superoxide dismutase 1 (SOD1) in a “neutrophil piggybacking” strategy. In the MCAO model, Neutrophil therapy delivered SOD1/CAT and Se to the site of brain injury in mice and significantly alleviated the area of cerebral infarction in mice after IS (Wang et al., 2022). In addition, the delivery of Se successfully upregulated GPX4 expression. At the same time, in addition to Neutrophil therapy, macrophages and monocytes also have this delivery capacity (Wang et al., 2022). Therefore, this neutrophil piggybacking strategy holds the promise of facilitating the application of nanomedicines in the central nervous system.

### 3.3 Relationship between nanoparticles-mediated ferroptosis and disease

The nanodelivery system is a sub-particulate carrier drug delivery system that modulates drug release rate and increases biofilm permeability. Numerous studies have reported that targeted ferroptosis nanomedicines can improve the low targeting and low solubility of conventional ferroptosis inhibitors (Żur and Farajpour, 2022). Also, several ferroptosis nanocarrier drugs have been used for ferroptosis-mediated diseases such as AKI, stroke, etc.

#### 3.3.1 Individual use of nanoparticles in ferroptosis-mediated disease

The injection of DFO in a model of Idiopathic pulmonary fibrosis (IPF) was found to increase survival (from 50% to 90%) and reverse the IPF phenotype in mice. However, low solubility and low targeting during drug delivery remain an insurmountable gap in DFO therapy. To solve this problem, the researchers prepared DFO nanomedicines and used a pulmonary drug-delivery system (PDDS) instead of oral administration or injection, and the biological effect of the drugs was significantly improved (Devkota et al., 2021).

The miR-134-5p inhibitor proposed by the investigators for the treatment of bronchopulmonary dysplasia (BPD) achieves a certain therapeutic effect, but it still suffers from the problems of conventional drugs such as drop targeting and low solubility. To better improve drug utilisation, the investigators further designed and synthesised the targeted ROS-responsive nanocarrier PCC-R8-ROS@miR-134-5p inhibitor (Lan et al., 2023), which more efficiently delivered the miR-134-5p inhibitor to the alveolar epithelial cells, providing a more efficient therapeutic strategy for BPD. However, in the future, improving encapsulation efficiency may remain a major challenge for delivering miRNAs.

AKI, a severe syndrome of renal insufficiency, was the focus of a study by Wang et al., 2021. They developed ultrasmall KCa(H_2_O)_2_ [FeIII(CN)_6_]-H_2_O nanoparticles, known as CaPB nano-enzymes, to function as a multienzymatic mimic. These nano-enzymes were effective in inhibiting ferroptosis by scavenging reactive oxygen/nitrogen species (RO/NSs) and treating AKI. The study found that CaPB nano-enzymes upregulated GPX4 both *in vivo* and *ex vivo* post intravenous administration, leading to kidney protection from oxidative damage and improved drug efficiency and targeting. Furthermore, the potential clinical application of CaPB nano-enzymes in AKI and other RO/NSs-related kidney diseases was highlighted. Xie et al., 2022 also contributed to the field by introducing gallic acid-gallium polyvinyl pyrrolidone nanoparticles (GGP NPs) as iron removers that could reduce intracellular free iron and mitochondrial dysfunction. These nanoparticles were able to downregulate iron-death-related substances like NADPH, GSH, GPX4, and ferritin, effectively suppressing ferroptosis-mediated AKI. Additionally, GGP NPs showed promise in ameliorating renal tubular injury and mitochondrial damage.

ICH remains an important cause of morbidity and mortality worldwide. Wang et al. presented a magnetically targeted nanocarrier loaded with the PPARγ agonist 15d-PGJ2-MNPs, which, when administered intravenously, activated PPARγ receptors on macrophages around haematomas, attenuated brain damage, and improved sensory and motor functions in mice after ICH (Wang et al., 2023). In addition, Yang et al. found that curcumin nanoparticles (Curcumin-NPs, Cur-NPs) could be effective in Cur delivery and provide ideas for the treatment of neurological impairment after ICH (Yang et al., 2021).

IS, a prevalent clinical neurological disorder, constitutes 85% of all strokes globally. Li et al., 2022 demonstrated that poly (lactic acid)-glycolic acid copolymerization-nanoparticles (SOD-PLGA-NPs) possess potent free radical scavenging capabilities, reducing cerebral infarct size in mice post-IS and ameliorating neurological deficits resulting from cerebral ischemia in the MCAO mouse model. Moreover, the nano delivery system enhances drug penetration through the blood-brain barrier.

Acute Liver Injury, a rare and life-threatening condition, was addressed by Shan et al. through the synthesis of ultrasmall poly (acrylic) acid coated Mn3O4 nanoparticles (PAA@Mn3O4-NPs, PMO), which effectively scavenge ROS, inhibit lipid peroxidation and ferroptosis, and mitigate Acute Liver Injury induced by acetaminophen and ischemia/reperfusion in mice. Furthermore, intravenous administration of PMO exhibits superior biocompatibility (Shan et al., 2023).

Myocardial Injury is a common complication of sepsis. Liu et al. designed and synthesised small biocompatible and MRI-visible melanin nanoparticles (MMPP) that could attenuate myocardial Injury. In *ex vivo* experiments, the investigators found that MMPP scavenges ROS and inhibits ferroptosis-mediated cardiomyocyte injury. In addition, MMPP downregulates oxidative stress induced by inflammatory factors (Alcalá-Alcalá et al., 2023).

Retinal pigment epithelial cells (RPE) are essential for maintaining the normal function and survival of photoreceptor cells. Oxidative stress and ferrous ion accumulation play a role in retinal degenerative diseases. Tang et al. found that a potent iron-conjugated nanoscale Prussian blue analogue, KCa [FeIII(CN)_6_] (CaPB), rescued retinal structure and visual function by preventing RPE degradation and was effective in preventing RPE lesions in mouse models (Tang et al., 2021).

#### 3.3.2 Targeted nanotechnology in combination with other drugs

The current use of targeted nanotechnology has to some extent improved the problems of traditional drug targeting, solubility, and time window. Recent studies have reported that targeted nanotechnology in combination with conventional drugs has improved drug targeting even further. In a rat model of cerebral ischemia-induced neurological impairment, the co-administration of tissue plasminogen activator (tPA) with nanoliposomal Fasudil-Lip showed better neuroprotection, and prolonged therapeutic time window (TTW) and improved drug targeting (Fukuta et al., 2017). Therefore, we can speculate whether ferroptosis inhibitors could also better address issues such as drug targeting by combining them with nanoliposome-encapsulated drugs.

## 4 Clinical diseases mediated by ferroptosis

Ferroptosis, a newly discovered form of cell death, has been found to have strong links with pathophysiological processes in neurological disorders, organ damage, and cardiovascular diseases.

AD is the most prevalent type of dementia, characterized by amyloid-β (Aβ) deposition in senile plaques (SPs) and intracellular neurofibrillary tangles (NFTs) formed due to hyperphosphorylation of tau proteins (Aggleton et al., 2016). Clinical research indicates that iron accumulation and oxidative stress are the primary pathological changes in the brains of AD patients. Aβ has been reported to convert intracellular iron to ferrous iron, leading to increased Aβ plaque formation, elevated ferritin expression, and worsening oxidative damage and cognitive impairments (Lane et al., 2023; Singh et al., 2024). Moreover, high levels of iron in the brain trigger the production of free radicals and induce oxidative stress. Studies have shown increased levels of HNE and acrolein, as well as upregulation of lipid peroxidation-related enzymes in the brains of individuals with AD. Additionally, reduced levels of GSH and inactivation of GPX4 have been observed in both animal models of AD and *postmortem* brain specimens (Yoo et al., 2010). Therefore, targeting ferroptosis inhibition could serve as a promising therapeutic approach for AD.

PD is characterized by the loss of dopaminergic neurons in the Substantia Nigra (SN) (Bae et al., 2021). The pathogenic mechanisms are multifaceted and involve α-synuclein accumulation, lipid peroxidation damage, iron deposition, oxidative stress, and inflammation. Among these, the production of lipid peroxides by α-synuclein within the cell membrane to induce ferroptosis plays a crucial role in the development of PD. Both iron accumulation and oxidative stress have long been linked to the progressive loss of dopaminergic neurons in PD patients, with dopaminergic neuron loss potentially further triggering ferroptosis (Thapa et al., 2022). Studies have shown that following the onset of PD, levels of DMT1 were elevated, leading to increased intracellular iron input, subsequently promoting dopaminergic neuronal death and α-syn accumulation. Additionally, a decrease in GSH levels post-PD activated 12-LOX, resulting in LOOH accumulation in the brain and worsening PD symptoms (Shibu et al., 2021). Furthermore, the use of ferroptosis inhibitors like DFP has shown promise in mitigating PD-related damage (Lin et al., 2022; Ding et al., 2023). Overall, exploring the relationship between ferroptosis and PD pathomechanisms may provide effective treatment options for patients with PD.

IS is associated with various pathological changes including ROS accumulation, ion metabolism disorders, and oxidative stress. Following cerebral ischemia, lipid peroxidation and iron accumulation are common characteristics of ferroptosis. Research has demonstrated that altered blood-brain barrier permeability post cerebral ischemia exacerbates ischemic injury, leading to disruptions in iron metabolism within brain tissues (Yang et al., 2022). This disruption induces the production of ROS through the Fenton reaction, along with an increase in ferritin, TFR1, and DMT1 expression in the brain. The accumulation of iron in the brain triggers ROS production via the Fenton reaction, worsening brain damage (DeGregorio-Rocasolano et al., 2019). Moreover, the brain contains high levels of unsaturated lipids that generate excess ROS, activating Nrf2 and p53, which further exacerbates oxidative stress and brain damage. Studies in rat MCAO models have shown elevated lipid peroxidation levels and reduced GSH levels (Millán et al., 2021). Thus, amelioration of neurologic impairment after IS may be facilitated by inhibiting relevant targets of ferroptosis.

SCI is a severe traumatic neurological disorder characterized by high levels of polyunsaturated fatty acids in the spinal cord, leading to oxidative stress. Studies have shown that rats with SCI exhibit iron overload in the motor cortex, resulting in the accumulation of Reactive Oxygen Species (ROS) and the induction of ferroptosis. Pathological changes such as iron deposition, lipid peroxide accumulation, and downregulation of GPX4 are commonly observed in both SCI patients and animal models (Wei et al., 2021). The activation of neuroglia after SCI results in the secretion of inflammatory factors, contributing to iron overload in the motor cortex (Li et al., 2023). Furthermore, the use of ferroptosis inhibitors like DFO and Fer-1 has been found to enhance motor function recovery following SCI (Li and Jia, 2023).

AKI is a common renal disease in clinical practice, and recent research suggests that ferroptosis may play a crucial role in IRI-AKI. During ischemia, AKI is exacerbated by elevated levels of free iron in the kidney, downregulation of GPX4 and SLC7A11, and depletion of blood GSH, leading to the activation of ferroptosis and subsequent tubular necrosis (Borawski and Malyszko, 2020). Zhao et al., 2020 demonstrated that iron deficiency worsened rhabdomyolysis (RM)-induced AKI. Additionally, ferroptosis inhibitors like Fer-1 and pioglitazone have been shown to reverse AKI damage in a mouse model by targeting ferroptosis, offering promising avenues for AKI treatment (Hosohata et al., 2022). Acute liver injury is another condition with high clinical mortality rates, and recent studies have highlighted the potential of Nrf2 activation in mitigating liver injury (Zhu et al., 2024). Furthermore, research by Yamada et al., 2020 indicated that upregulation of ferroptosis markers (iron ions, lipid peroxide levels) exacerbated liver injury in hepatic IRI models, but treatment with ferroptosis inhibitors like Fer-1 significantly reduced injury severity.

Studies have shown that ferroptosis is associated with MIRI, heart failure, atherosclerosis, myocardial infarction, and other cardiovascular diseases. Iron metabolism and lipid metabolism play important roles in the regulation of cardiovascular disease. Of these, iron ion disorders are the most common. TFR1 expression was found to be upregulated during MIRI (Miyamoto et al., 2022). In contrast, TFR1 inhibitors such as DMT1i are protective against ferroptosis-mediated heart disease. In cardiovascular disease, LOX expression is upregulated, which in turn promotes lipid peroxidation and ferroptosis (Fratta Pasini et al., 2023). Meanwhile, inhibition of ACSL4 improves cardiac function and prevents heart failure (Ito et al., 2021). Furthermore, Upregulation of SLC7A11 and GPX4 Downregulates the Mouse Lipid Peroxidation and Iron Levels in the Endothelium of Mouse Aorta and Attenuates Atherosclerotic Injury (Bai et al., 2020).

## 5 Challenges encountered in the clinical translation of ferroptosis inhibitors

There have always been several problems in the development of drugs. In general, new drugs tend to face many difficulties and challenges before they are launched on the market. Since ferroptosis was proposed, the development of ferroptosis inhibitors as well as their clinical application has also become a topic of increasing interest for researchers. Although many small-molecule ferroptosis inhibitors have been developed and continuously optimised, most of them are still difficult to use successfully in the clinic.

### 5.1 Challenges encountered in preclinical trials

Several drugs have been shown to inhibit ferroptosis in animal experiments, but they mostly suffer from low stability, low solubility, low targeting, low safety, toxicity, poor pharmacokinetics, and low activity (Guo et al., 2023), so very few of them have entered clinical studies. For example, **Fer-1**, the first synthetic inhibitor of ferroptosis, has been shown to play a role in many diseases in animal models (Skouta et al., 2014; Wang et al., 2023), but is still in the experimental stage, making it difficult to cross over to clinical trials. Mainly due to its low stability and low solubility, the researchers designed and synthesised **compound 39** based on **Fer-1**, **compound 39** can significantly improve its ferroptosis inhibition potency (Linkermann et al., 2014; Scarpellini et al., 2023). Meanwhile, phenothiazine-based derivatives **compound 51**, although it has shown neuroprotective effects in IS animal models, still has high hERG inhibitory activity (Yang et al., 2021; You et al., 2022). **Se** has long been shown to alleviate diseases such as iron-death-mediated stroke by enhancing adaptive transcription, but Ishraq Alim et al. found a parabolic dose curve after **Se** supplementation *in vitro* and *in vivo* experiments, raising concerns about the mode of **Se** delivery as well as its safety. Moreover, **Se**-mediated GPX4 regulation and neuronal protection are extremely complex and therefore difficult to successfully translate into the clinic (Alim et al., 2019). Although **DFO** reversed the survival of IPF mice, it still suffers from low solubility and targeting during delivery (Guo et al., 2023). In addition, the natural substance **RES** is still in the preclinical experimental stage and is difficult to translate because of its problems such as low water solubility, easy degradation, low activity, and poor pharmacokinetics (Pharmacokinetics, PK) (Chung et al., 2020; Ni et al., 2023). Therefore, there is a need for further improvement of the drug thus better marketing of the drug to clinical studies.

### 5.2 Challenges encountered in clinical studies

After a drug has gone through the preclinical phase of experimentation, the drug needs to be tested in clinical trials (Moujalled et al., 2021; Iasonos and OQuigley, 2021). Several ferroptosis inhibitors have also made it to the clinical translational stage in recent years, but they still present challenges in clinical studies.

**Vitamin E** was found to significantly reduce cognitive performance in patients with mild to moderate AD in a multicentre, randomised, double-blind, placebo-controlled trial of AD from 2007 to 2012. However, some studies claim that **vitamin E** supplementation does not reduce the risk of AD and slows down its pathogenesis (Dysken et al., 2014; Kryscio et al., 2017; Ashraf and So, 2020).

In 2013, **DFP** significantly improved motor function in patients with PD in a 12-month phase II clinical trial of PD, but patients who continued to take **DFP** showed diminished improvement in motor function as well as reduced Neutrophils and granulocyte deficiencies (Devos et al., 2014; Martin-Bastida et al., 2017). In addition, Mohsen Saleh Elalfy et al. conducted a clinical trial on **DFP** for the treatment of transfusion-dependent thalassemia children in 2016. They found that the safety and efficacy of the drug was improved when **DFP** was administered to thalassemia patients, but adverse effects such as diarrhea, vomiting, colic, neutropenia, and elevated liver enzymes were still present (Elalfy et al., 2018).

In 2019, an injection of **Edaravone dexborneol** in phase II multicentre, randomised, double-blind, multi-dose, active-controlled clinical trial in patients with IS found that patients experienced a reduction in acute brain injury, but continued to have itching and AKI side effects (Xu et al., 2019).

Copper compound **CuII(ATSM)** exhibits neuroprotective effects in phase I clinical trials in ALS and PD (Southon et al., 2020). Meanwhile, **CuII (ATSM)** has completed phase I trials in ALS patients and entered phase II clinical trials, but it has been found to exhibit spinal cord immunocompromise in patients in the clinical trial phase (Liddell et al., 2023).

In recent years, it has been found that ferroptosis-mediated neurological impairment can be significantly alleviated by thrombin inhibition. Meanwhile, the thrombin inhibitor **Dabigatran** is currently in Phase III clinical trial in IS (NCT03961334) (Tuo et al., 2022). In April 2023, the thrombin inhibitor **Argatroban** concluded a Phase IV clinical trial in IS (NCT03740958) and was found to significantly reduce adverse prognosis when used in combination with Aspirin (Peng, 2023).

Therefore, there is a need for researchers to further optimise drugs to improve drug utilisation, reduce side effects and treat ferroptosis-mediated diseases.

## 6 Ferroptosis in relation to other modes of cell death

Ferroptosis is a type of programmed cell death that is distinguished from apoptosis, necrotic apoptosis, autophagy, pyroptosis and cuproptosis in both morphological changes and biochemical functions. However, a growing body of research suggests that there is a link between ferroptosis and all of the above modes of cell death.

### 6.1 Ferroptosis and apoptosis

Apoptosis, the first identified regulatory cell death modality, is governed by apoptosis-related genes. The initiation of apoptosis is often characterized by morphological alterations such as cell shrinkage, chromatin fragmentation, and membrane blistering and disintegration. Apoptosis encompasses caspase-dependent intrinsic apoptosis, death receptor-mediated extrinsic apoptosis, and receptor-dependent extrinsic apoptosis. Research indicates that Tumor Necrosis Factor-α (TNF-α) plays a crucial role as an inflammatory factor that triggers ferroptosis. Additionally, Kai et al. (2020) reported the induction of chondrocyte apoptosis through the upregulation of TNF-α (Yang et al., 2021). Furthermore, Ma et al., 2022 demonstrated that Fer-1 mitigates noise-induced hearing loss by targeting TFR1-mediated ferroptosis and the p53-AIFM2 apoptosis pathway.

### 6.2 Ferroptosis and necroptosis

Necroptosis is a type of regulatory necrosis that is often caused by a variety of physicochemical stimuli, such as inflammatory factors and ATP depletion and is not dependent on caspases. Necrotic apoptosis is followed by morphological changes such as rupture of cell membranes, translucent cytoplasm, swollen organelles, increased cell volume, and chromatin condensation. Iron overload was found to affect cellular redox homeostasis and activate necrotic apoptosis and ferroptosis. In recent years, the ferroptosis inhibitor Lip-1 was found to be effective in inhibiting necrotic apoptosis during cerebral ischemia-reperfusion. Meanwhile, the necrotic apoptosis inhibitor Necrostatin-1 effectively inhibited the activation of proteins associated with ferroptosis (Yuk et al., 2021). In addition, Nigratine (also known as 6E11) was found to inhibit both necrotic apoptosis and ferroptosis in human bronchial-like organs (Delehouzé et al., 2022). Thus, ferroptosis and apoptosis have complex interactions and are closely related.

### 6.3 Ferroptosis and autophagy

Autophagy is the process of removing and degrading intracellular material through lysosomes, which includes macroautophagy, microautophagy, and chaperone-mediated autophagy. This process plays a vital role in maintaining cellular homeostasis and preventing cell overgrowth. Studies have shown a close relationship between ferroptosis and ferritin autophagy, adipose autophagy, and chaperone-mediated autophagy (Lee et al., 2023). Moreover, ROS accumulation, p53 activation, and Nrf2 signaling pathway activation all induced autophagy and ferroptosis. Autophagy-dependent ferroptosis has been implicated in kidney-related diseases and cardiovascular diseases. Additionally, research by Du et al., 2023 demonstrated that rapamycin injection enhanced autophagy, leading to the downregulation of TFR1, which inhibited ferroptosis and reduced cognitive deficits in a Sepsis-associated encephalopathy (SAE) model in mice.

### 6.4 Ferroptosis and pyroptosis

Pyroptosis, also known as cellular inflammatory necrosis, is programmed cell death induced by activation of inflammatory vesicles and is mainly regulated by cysteinyl asparagine-1, -4, -5, and -11-dependent signaling pathways (Teng et al., 2023). Pyroptosis was accompanied by morphological changes such as cell swelling, membrane rupture, and leakage of cytoplasmic components. It has been shown that high intracellular ROS accumulation promotes pyroptosis and that severe oxidative stress induces upregulation of pyroptosis. In recent years, it has been found that a mixed form of cell death, “PAN apoptosis”, consisting of TNF-α and interferon gamma (IFNγ) or Proadrenomedullin N-terminal 20 peptide (PAMP), can activate pyroptosis and apoptosis at the same time. IFNγ can form an important anti-ferroptosis system with SLC3A2 (Ai et al., 2024).

### 6.5 Ferroptosis and cuproptosis

Cuproptosis is defined as the aggregation of proteins, proteotoxic stress, and eventual cell death triggered by copper binding to the lipolytic enzymes of the tricarboxylic acid (TCA) cycle. This form of regulated cell death, dependent on copper, was termed “copper tumor” in 2022, and has been linked to the metabolic pathways of ferroptosis. Similar to ferroptosis, cuproptosis leads to notable alterations in mitochondrial structure, such as shrinkage and membrane rupture. The mitochondrial TCA cycle serves as a crucial nexus between ferroptosis and copper tumors, playing a pivotal role in cuproptosis (Jhelum and David, 2022). Furthermore, GSH has been identified as a key player in both ferroptosis and cuproptosis. GSH binds copper, reducing protein aggregation in cuproptosis. Moreover, iron-sulfur cluster proteins, essential cofactors for maintaining redox balance and iron levels, are produced as auxiliary enzymes in cuproptosis, with their levels significantly decreasing following cuproptosis initiation. Notably, protein aggregates can interfere with the function of iron-sulfur clusters (Prasad Panda and Kesharwani, 2023).

## 7 Discussion and conclusion

Ferroptosis inhibitors have displayed some efficacy in ferroptosis-related diseases, yet face significant challenges in making a substantial impact. These obstacles stem from the limitations of current small molecule drugs, characterized by poor selectivity, targeting, solubility, pharmacokinetic properties, adverse reactions, efficacy, *in vivo* toxicity, and side effects. On the other hand, some of the ferroptosis mechanisms themselves are unclear, and some of the mechanisms have been proposed but are still unknown (e.g., GCH1-BH4 pathway, HSF1-HSPB1 pathway, sulfur-transfer pathway, MVA pathway, etc.), which has become an important obstacle in the development of small molecule drugs. In addition, the complexity of the pathological mechanisms of some diseases is unclear, and the controversial relationship between ferroptosis and other modes of cell death is also an issue that needs to be addressed for the application of ferroptosis inhibitors to disease.

Overall, this review specifically describes the metabolic pathways of ferroptosis. A comprehensive summary of natural and synthetic small molecule drugs that treat ferroptosis-mediated diseases by acting on targets of ferroptosis and their therapeutic efficacy in disease. Meanwhile, this paper describes the mechanism of action of therapeutic approaches such as gene regulatory approaches, cell therapy, and nanodelivery in ferroptosis-mediated diseases. And it summarizes the problems encountered during the clinical translation of ferroptosis inhibitors and drugs that are currently in clinical translation. Finally, the relationship between ferroptosis and other forms of cell death modalities such as apoptosis is discussed. It is hoped that this will provide new ideas for the development of future ferroptosis inhibitors and provide effective therapeutic options for future ferroptosis-mediated diseases.
